# Self-management intervention to reduce pulmonary exacerbations by supporting treatment adherence in adults with cystic fibrosis: a randomised controlled trial

**DOI:** 10.1136/thoraxjnl-2021-217594

**Published:** 2021-09-23

**Authors:** Martin J Wildman, Alicia O’Cathain, Chin Maguire, Madelynne A Arden, Marlene Hutchings, Judy Bradley, Stephen J Walters, Pauline Whelan, John Ainsworth, Iain Buchan, Laura Mandefield, Laura Sutton, Paul Tappenden, Rachel A Elliott, Zhe Hui Hoo, Sarah J Drabble, Daniel Beever, CFHealthHub Study Team, H Cantrill

**Affiliations:** 1 Sheffield Adult Cystic Fibrosis Centre, Sheffield Teaching Hospitals NHS Foundation Trust, Sheffield, UK; 2 School of Health and Related Research, University of Sheffield, Sheffield, UK; 3 Clinical Trials Research Unit, University of Sheffield, Sheffield, UK; 4 Centre for Behavioural Science and Applied Psychology, Sheffield Hallam University, Sheffield, UK; 5 Wellcome-Wolfson Institute for Experimental Medicine, School of Medicine Dentistry and Biomedical Sciences, Queen's University Belfast, Belfast, UK; 6 Health eResearch Centre – Division of Imaging, Informatics and Data Sciences, Faculty of Biology, Medicine and Health, The University of Manchester School of Health Sciences, Manchester, UK; 7 Department of Public Health and Policy, Institute of Population Health, University of Liverpool, Liverpool, UK; 8 Division of Population Health, Health Services Research and Primary Care, Faculty of Biology, Medicine and Health, The University of Manchester School of Health Sciences, Manchester, UK

**Keywords:** cystic fibrosis, psychology, nebuliser therapy

## Abstract

**Introduction:**

Recurrent pulmonary exacerbations lead to progressive lung damage in cystic fibrosis (CF). Inhaled medications (mucoactive agents and antibiotics) help prevent exacerbations, but objectively measured adherence is low. We investigated whether a multi-component (complex) self-management intervention to support adherence would reduce exacerbation rates over 12 months.

**Methods:**

Between October 2017 and May 2018, adults with CF (aged ≥16 years; 19 UK centres) were randomised to the intervention (data-logging nebulisers, a digital platform and behavioural change sessions with trained clinical interventionists) or usual care (data-logging nebulisers). Outcomes included pulmonary exacerbations (primary outcome), objectively measured adherence, body mass index (BMI), lung function (FEV_1_) and Cystic Fibrosis Questionnaire-Revised (CFQ-R). Analyses were by intent to treat over 12 months.

**Results:**

Among intervention (n=304) and usual care (n=303) participants (51% female, median age 31 years), 88% completed 12-month follow-up. Mean exacerbation rate was 1.63/year with intervention and 1.77/year with usual care (adjusted ratio 0.96; 95% CI 0.83 to 1.12; p=0.64). Adjusted mean differences (95% CI) were in favour of the intervention versus usual care for objectively measured adherence (9.5% (8.6% to 10.4%)) and BMI (0.3 (0.1 to 0.6) kg/m^2^), with no difference for %FEV_1_ (1.4 (−0.2 to 3.0)). Seven CFQ-R subscales showed no between-group difference, but treatment burden reduced for the intervention (3.9 (1.2 to 6.7) points). No intervention-related serious adverse events occurred.

**Conclusions:**

While pulmonary exacerbations and FEV_1_ did not show statistically significant differences, the intervention achieved higher objectively measured adherence versus usual care. The adherence difference might be inadequate to influence exacerbations, though higher BMI and lower perceived CF treatment burden were observed.

**Key messages**

**What is the key question?**

- Can a multi-component self-management intervention increase and sustain adherence to inhaled therapies among adults with cystic fibrosis (CF) and does the intervention impact on exacerbation rates?

**What is the bottom line?**

- The intervention did not show a statistically significant difference in exacerbation rates versus usual care but achieved higher objectively measured adherence to inhaled medications (sustained over 12 months), higher body mass index and lower perceived CF treatment burden.

**Why read on?**

- This is the largest self-management intervention trial in CF, with 607 participants, and the only trial thus far to demonstrate a sustained difference in adherence versus a control arm, using a theory-based approach including habit formation.

## Introduction

Cystic fibrosis (CF) is a multisystem genetic long-term condition (LTC) whereby recurrent pulmonary exacerbations drive progressive lung damage leading to premature death. Inhaled mucoactive agents and antibiotics have proven efficacy in reducing exacerbation frequency.^1 2^ CF is therefore an archetypal LTC; a cure is unavailable though efficacious treatments exist to improve health outcomes.

Low medication adherence, described by the WHO as ‘a worldwide problem of striking magnitude’^3^ is an important cause of treatment failure, poor health outcomes and increased healthcare costs in LTCs. In CF, low adherence to inhaled therapies is associated with more frequent and costly rescue treatments of exacerbations.^4^ Real-world objectively measured adherence of 30%–50% is lower than that of 80%–100% usually observed in clinical trials.^5^ Therefore, people with CF are unlikely to derive optimal benefit from inhaled therapies.^5–10^ At the same time, perceived treatment burden is high among people with CF^11^ and the James Lind Alliance Priority Setting Partnership identified ‘effective ways of simplifying treatment burden’ as the top CF research priority.^12^

Developing strategies to increase adherence is another CF research priority^12^ and could improve health outcomes by reducing exacerbation rates.^6^ Insufficient evidence exists to promote any particular adherence strategy.^13^ Large randomised controlled trials evaluating adherence interventions in CF continue to present negative findings,^14^ possibly because interventions are generally under-theorised and insufficiently tailored to individual needs.^15^ In addition, objectively measured adherence is largely absent in routine CF care, while self-reported and clinician estimates of adherence are notoriously unreliable,^8^ which prevents effective diagnosis, prescribing and provision of person-specific adherence support.

The Capability Opportunity Motivation-Behaviour model, based on a synthesis of frameworks of behavioural change, predicts that treatment taking depends on capability, opportunity and motivation.^16^ Reflective motivation is largely dependent on perceived necessity of adherence and treatment concerns^17^ and can be increased through education, persuasion and confidence building. For those motivated to adhere, increasing awareness of their objectively measured adherence through self-monitoring increases capability, that is, making visible the gap between objective and subjective adherence.^8^ Problem-solving techniques can be used to overcome individual capability and opportunity barriers. Theories of behavioural maintenance^18^ predict that supporting people to create habits for treatment, that is, taking treatments in response to specific contextual cues, can help to sustain adherence and to lower perceived treatment burden.^19^ We developed a multi-component (complex) self-management intervention to support sustained treatment adherence,^20^ incorporating objective adherence measurement, underpinned by behavioural science theory and designed to address gaps in CF care, with extensive input from people with CF. Since exacerbations are disruptive to patient life, they are an important patient-centred outcome and are commonly considered to indicate lung health.^21^ Therefore, the objective of this 12-month randomised controlled trial was to investigate the effectiveness of this multi-component self-management intervention compared with usual care in adults with CF using pulmonary exacerbation incidence rate as the primary outcome.

## Methods

### Study design, clinical interventionists and participants

We conducted a two-arm, open-label, parallel-group, usual care-controlled randomised clinical trial at 19 UK CF centres (trial registration ISRCTN55504164). The protocol (ethical approval REC: 17/LO/0035, IRAS ID: 218519) and statistical analysis plan (SAP) are available as supplementary material. The complex behavioural change intervention is designed to increase and sustain adherence to inhaled therapies. The development of the intervention is described elsewhere.^22^ Table 1 summarises the intervention, and further descriptions are in online supplemental appendix A. The study was monitored by an independent Trial Steering Committee. Data analysis was performed by the School of Health and Related Research, Sheffield. All authors vouch for fidelity to the protocol.

10.1136/thoraxjnl-2021-217594.supp1 Supplementary data

**Table 1:** Description of the intervention

TIDieR category	Description of the CFHealthHub intervention
	CFHealthHub digital platform (website and smartphone application) that: (1) displays real-time objective adherence data from the nebulisers to the participant and care team, (2) provides behavioural change tools and content (comprises of six modules, see 1)) in a ‘My Toolkit’ area designed to increase motivation for adherence, to address capability and opportunity barriers and to build habits for taking treatments, and (3) includes an intervention manual, with procedures and worksheets for use by clinical interventionists in their interactions with participants. Behaviour change sessions where the content and behaviour change techniques within each of the six modules were delivered through the participant interactions with site’s interventionist.
Why	CFHealthHub aims to support adults with CF to increase adherence to nebuliser treatment using the COM-B framework and to build habits for treatment to enable maintenance.
Who	Interventionists were healthcare professionals employed for the trial (n=32), 18 of whom job shared (with clinical roles in the CF team) and 26 were existing members of the centre’s CF multidisciplinary team. There was the WTE of eight physiotherapists, three psychologists, six specialist CF nurses, one pharmacist and one dietitian; that is, one WTE interventionist per centre.
How and where	All intervention sessions were structured by a worksheet to guide delivery and delivered with a person-centred communication style. First intervention sessions were always face to face; review sessions were face to face or by telephone.
When and how much	Intervention participants had access to the digital platform and received tailored flexible support from the interventionist throughout the 12-month trial period. All intervention participants received a first and intermediate review visit, thereafter support was tailored according to response (figure 1; further details in online supplemental 1appendix A). Participants with baseline objectively measured effective adherence ≤80% underwent a normal pathway of six sessions (1× first intervention visit 40–60 min; 2× intermediate reviews 5–15 min; 2× reviews 30–45 min; 1× phase review 20–30 min) over 12 weeks, with phase reviews every 12 weeks thereafter, or every 6 weeks for participants with baseline adherence <25%. Participants with baseline objectively measured effective adherence >80% followed a ‘very high adherence’ pathway of three sessions (1× first intervention visit; 1× intermediate review; 1× phase review), with phase reviews every 12 weeks thereafter. Following these initial pathways, additional blocks of sessions were offered when: (1) a participant requested further support; (2) a participant’s adherence reduced by ≥20% in a 4-week period; or (3) a participant received intravenous antibiotics for an exacerbation.
Tailoring	Each session was tailored to an individual’s needs based on: their nebulised medication prescription; their necessity and concern beliefs (BMQ-Specific); and their discussions with interventionists about their motivation and specific capability and opportunity barriers to adherence. For example, the goal setting and review and treatment plan modules are used only for participants who are motivated to increase their treatment adherence and participants with very low motivation spend more time focusing on the my treatment module and on relationship building with the interventionist. While the entire content of the digital platform was available for participants to browse, tailored/personalised aspects were added to the ‘My Toolkit’ area. For example, content addressing particular participant concerns about treatment, and personal action and coping plans.
Modifications	There were no major changes to the delivery of the intervention through the study.
How well	Fidelity of intervention delivery was assessed throughout the study with two reviewers independently assessing a sample of audio-recording and worksheets from sessions (first intervention session, review, phase review) using a scoring sheet (further details in online supplemental appendix A).

BMQ-Specific, beliefs and medications questionnaire-specific; CF, cystic fibrosis; COM-B, capability opportunity motivation-behaviour; TIDieR, template for intervention description and replication; WTE, whole time equivalent.

Full-time interventionists (table 1) were employed and trained to deliver the intervention, underwent competency assessments (a theory test; practical assessments at first intervention visit, review and phase review) and received ongoing support (as detailed in online supplemental appendix A).

Participants were identified from the CF Registry, a UK database of people with CF. Potential participants were contacted by their usual clinical care team to seek permission for interventionists to discuss involvement. Eligible participants were aged ≥16 years and willing to take all inhaled mucoactive agents and antibiotics via eFlow Technology nebulisers with eTrack data-logging Controllers (PARI Pharma GmbH, Starnberg, Germany). Participants were excluded if: on the active lung transplant list; post-lung transplant; receiving care primarily palliative in intent; or using inhaled dry powder devices. All participants provided written, informed consent.

### Randomisation and masking

Participants were allocated 1:1 to the intervention or usual care using a computer-generated pseudorandom list with random-permuted blocks of randomly varying sizes, via a central, web-based randomisation system. The allocation sequence was hosted by the Sheffield Clinical Trials Research Unit, with the sequence created by a statistician (not otherwise involved with trial) and held on a secure server. After recruiting each participant, the interventionist logged into the server and entered basic demographic information, then the allocation was revealed to the participants. Stratification was by centre and number of past year intravenous antibiotic days (≤14 and >14) – a predictor of current year intravenous days.^23^ The trial statistician remained blind to treatment allocation until database freeze. Participants and health professionals collecting primary outcome data were not blinded.

### Treatment arms

All participants were given eTrack data-logging Controllers for their eFlow Technology nebulisers, which sent time-stamped and date-stamped data to a 2net Hub (Capsule Technologies, San Diego, California, USA) for accurate recording of inhalation and adherence calculation.

**Figure 1:**
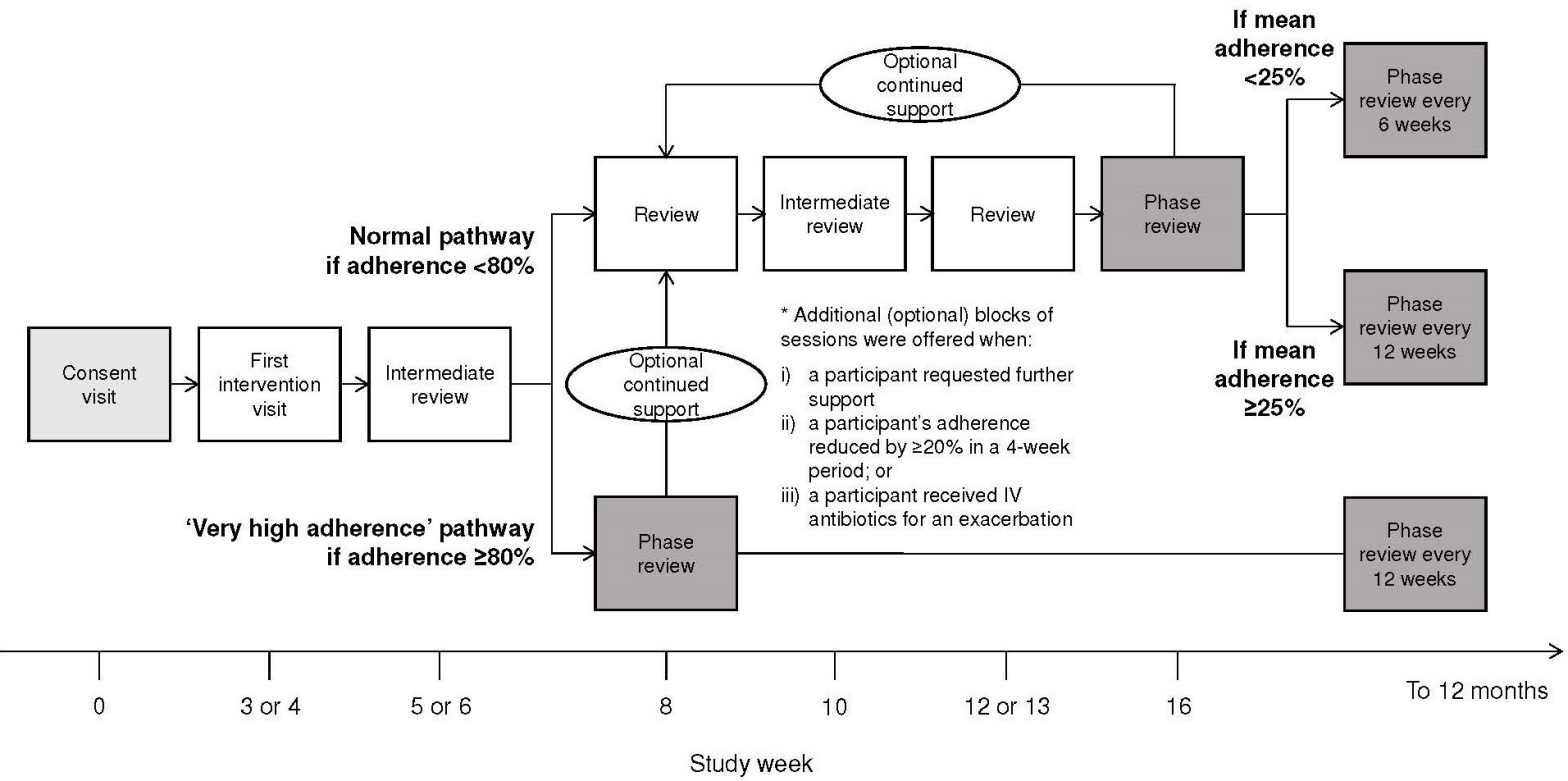
Schedule of intervention delivery: normal and ‘very high adherence’ pathways. Adherence level to reflect baseline was calculated using objectively measured effective adherence data from weeks 1 and 2, as stated in the ‘Methods’.

Intervention participants had access to the CFHealthHub digital platform (website and smartphone application) and received tailored flexible support from the interventionist throughout the 12-month trial period (table 1 and figure 1; further details in online supplemental appendix A).

The usual care arm used eTrack data-logging Controllers for adherence data collection. Contamination was minimised since there was no access to CFHealthHub, behavioural change tools and content. Adherence results were also invisible to participants and care teams.

At the final visit (at 12 months) or when a participant dropped out in either arm, a systems check was performed to ensure that all adherence data had been transferred from the eTrack data-logging Controller, thereby minimising missing data. The eTrack can store 3000 inhalations, which exceed the annual total number of doses even when the maximum prescription of eight daily doses occurred (table 2 gives prescribed doses), ensuring no missing data if eTrack was downloaded at the end of trial.

**Table 2:** Baseline demographic and clinical characteristics, by randomised treatment group

	Usual care		Intervention	
	N*	Mean±SD†	N*	Mean±SD†
Female, n (%)	303	154 (50.8)	304	156 (51.3)
Age, years	303	30.3±10.8	304	31.1±10.6
Prescribed number of daily nebuliser doses, n (%)				
1	298	60 (20.1)	303	85 (28.1)
2	298	49 (16.4)	303	39 (12.9)
3	298	93 (31.2)	303	91 (30.0)
4	298	38 (12.8)	303	32 (10.6)
5	298	38 (12.8)	303	3 (10.9)
6	298	9 (3.0)	303	10 (3.3)
≥7	298	11 (3.7)	303	13 (4.3)
Socioeconomic deprivation quintiles, n (%)				
1 (least deprived)	302	51 (16.9)	302	50 (16.6)
2	302	71 (23.5)	302	59 (19.5)
3	302	66 (21.9)	302	63 (20.9)
4	302	67 (22.2)	302	63 (20.9)
5 (most deprived)	302	47 (15.6)	302	67 (22.2)
*Pseudomonas aeruginosa* status, n (%)‡				
Chronic	299	175 (58.5)	304	174 (57.2)
Non-chronic	299	124 (41.5)	304	130 (42.8)
Previous year’s intravenous treatment, days	303	27.7±33.0	304	24.2±27.9
Secondary outcomes: baseline values				
Objectively measured effective adherence (weekly), %§	295	45.5±34.1	293	54.1±33.0
FEV_1_ % predicted	302	58.3±22.6	304	60.7±23.5
Body mass index, kg/m^2^	303	22.5±4.2	304	22.7±4.2
Patient-reported outcomes: baseline values				
CFQ-R (quality of life):				
Physical	302	53.0±30.2	304	54.3±30.6
Emotional	302	66.2±24.1	304	66.5±21.6
Social	302	60.9±20.9	304	61.9±20.0
Eating	302	80.5±24.3	304	82.1±22.5
Body image	302	66.1±29.3	304	65.6±28.0
Treatment burden	302	51.8±20.2	304	54.4±19.8
Respiratory	302	56.6±21.9	304	58.2±22.1
Digestion	302	81.1±19.4	304	79.9±21.5
BMQ-Specific (beliefs about medication):				
Concerns	301	2.1±0.5	304	2.1±0.6
Necessities	301	3.6±0.8	304	3.6±0.7
SRBAI (habit strength for using nebuliser)	300	12.0±4.7	303	12.1±5.0
Perceptions of treatment adherence (three-item scale)	274	9.9±3.4	280	10.2±3.4
Effort of nebuliser treatments (one item)	300	3.1±1.2	302	3.1±1.3
Subjective adherence question – % (self-report estimate of adherence)	298	69.0±30.8	300	69.9±31.0
CHAOS-6 (life chaos or routine)	300	9.5±2.9	303	9.5±2.9
PAM-13 (health style assessment)	302	65.3±13.3	304	65.8±14.5
EQ-5D-5L (generic health status)	300	0.84±0.16	303	0.85±0.15
PHQ-8 (depression)	301	6.4±5.1	304	6.4±5.2
GAD-7 (anxiety)	302	4.7±4.7	302	4.6±4.9

Full details and references for all patient-reported outcomes are available in the SAP (provided in online supplemental material).

*There were 608 participants randomised but one participant randomised to the intervention arm withdrew on the day of consent prior to baseline data collection, giving a maximum n=607 for baseline summaries.

†Unless otherwise stated.

‡Consensus definition.

§Weekly objectively measured effective adherence (sum of doses taken/sum of doses prescribed).

¶All patient-reported outcomes based on points, unless otherwise stated. For direction of positive effect and possible range, see table 3.

BMQ, Beliefs About Medicines Questionnaire; CHAOS-6, Confusion, Hubbub and Order six-item Scale; EQ-5D-5L, EuroQol 5-dimension and 5-level; GAD-7, Generalised Anxiety Disorder seven-item scale; PAM-13, Patient Activation 13-item Measure; PHQ-8, Patient Health Questionnaire eight-item depression scale; SAP, statistical analysis plan; SRBAI, Self-Report Behavioural Automaticity Index.

### Outcomes

The primary outcome was the pulmonary exacerbation incidence rate over 12 months. Using modified Fuchs’ criteria,^24^ an exacerbation occurred if intravenous antibiotics were administered for any one of 12 prespecified symptoms. Exacerbation forms were completed by a healthcare professional (clinical team or interventionist), and documented assessments were conducted at each clinical encounter (generally every 3 months) determined whether a participant was displaying an exacerbation.

To reflect effective medication use, adherence was calculated as normative (effective) adherence^9 10^ using objective data from weeks 3–52 as the outcome and weeks 1 and 2 as the ‘baseline’. Objectively measured effective adherence was calculated daily as a composite of all inhaled medications then aggregated weekly for analysis (appendix B), as we have detailed elsewhere.^9 10^ Other secondary endpoints were percent predicted FEV_1_), measured at each clinical encounter, and body mass index (BMI), calculated at baseline and 12 months. Patient-reported outcomes collected at baseline and 12 months included: CF Questionnaire-Revised (CFQ-R; eight subscales), measuring quality of life and including a perceived CF treatment burden subscale; Beliefs About Medicines Questionnaire (BMQ)-specific concerns and necessities; Self-Report Behavioural Automaticity Index (SRBAI), measuring habit strength; perceptions of treatment adherence (three-item scale); perceived effort of nebuliser treatments (one item); subjective adherence (self-reported % adherence); Confusion, Hubbub and Order 6-item Scale (CHAOS-6); Patient Activation 13-item Measure (PAM-13); and EuroQol 5-dimension and 5-level generic health status. Patient Health Questionnaire eight-item depression scale and Generalised Anxiety Disorder seven-item scale (GAD-7) were safety measures to understand whether the intervention worsens depression or anxiety. Adverse events were recorded using case report forms and were categorised as whether or not expected in relation to medications, or common among people with CF. Full details and references for outcomes are in the protocol and SAP.

### Statistical analysis

Power calculations (online supplemental table 1) informed the choice of pulmonary exacerbation as the primary outcome and individual (vs cluster) randomisation. Cluster trials are complicated by recruitment bias. While contamination that reduces effect size may be a risk with individual randomisation, this can usually be overcome by increasing the sample size, which often still requires a smaller sample than cluster randomisation.^25^ The sample size was predicted based on reducing two exacerbations per year to 1.5 per year (equivalent to an incidence rate ratio of 2.0/1.5=0.75). Assuming a mean difference of 0.5 pulmonary exacerbations between the intervention and usual care arms over 12 months, an SD of 1.5, a design effect of 1.16 to allow for any clustering of outcomes by centre (intraclass correlation 0.01; cluster size 17) and an attrition rate of 20%, 556 participants were required to provide 90% power at a two-sided 5% level of significance.

Baseline characteristics were reported descriptively using summary statistics. The primary outcome incidence rate ratio, 95% CI and p value were estimated using a negative binomial regression model, with a random effect to adjust for clustering by centre. Log follow-up time was an offset in the model, and past year intravenous days (≤14 and >14 days) and treatment arm were fixed effects. Details of the sensitivity analyses performed on the primary outcome data (including adjustment for missing data) are in the SAP. Objectively measured effective adherence was analysed using a linear mixed-effects model, with random slopes and intercepts; treatment arm, time in weeks, baseline adherence (measured in the first 2 weeks post randomisation) and past year intravenous days were fixed effects. Treatment effects and 95% CI for all other secondary outcomes were produced using a mixed-effects model adjusting for baseline and past year intravenous days and with a random effect to adjust for clustering by centre. To aid interpretation, standardised effect sizes (Cohen’s d) were calculated for all secondary outcomes by dividing treatment effect with pooled SD

All analyses were prespecified and performed by intent to treat using R software V.3.6.1 and SAS V.9.4. CI widths were not corrected for multiplicity.

## Results

**Figure 2:**
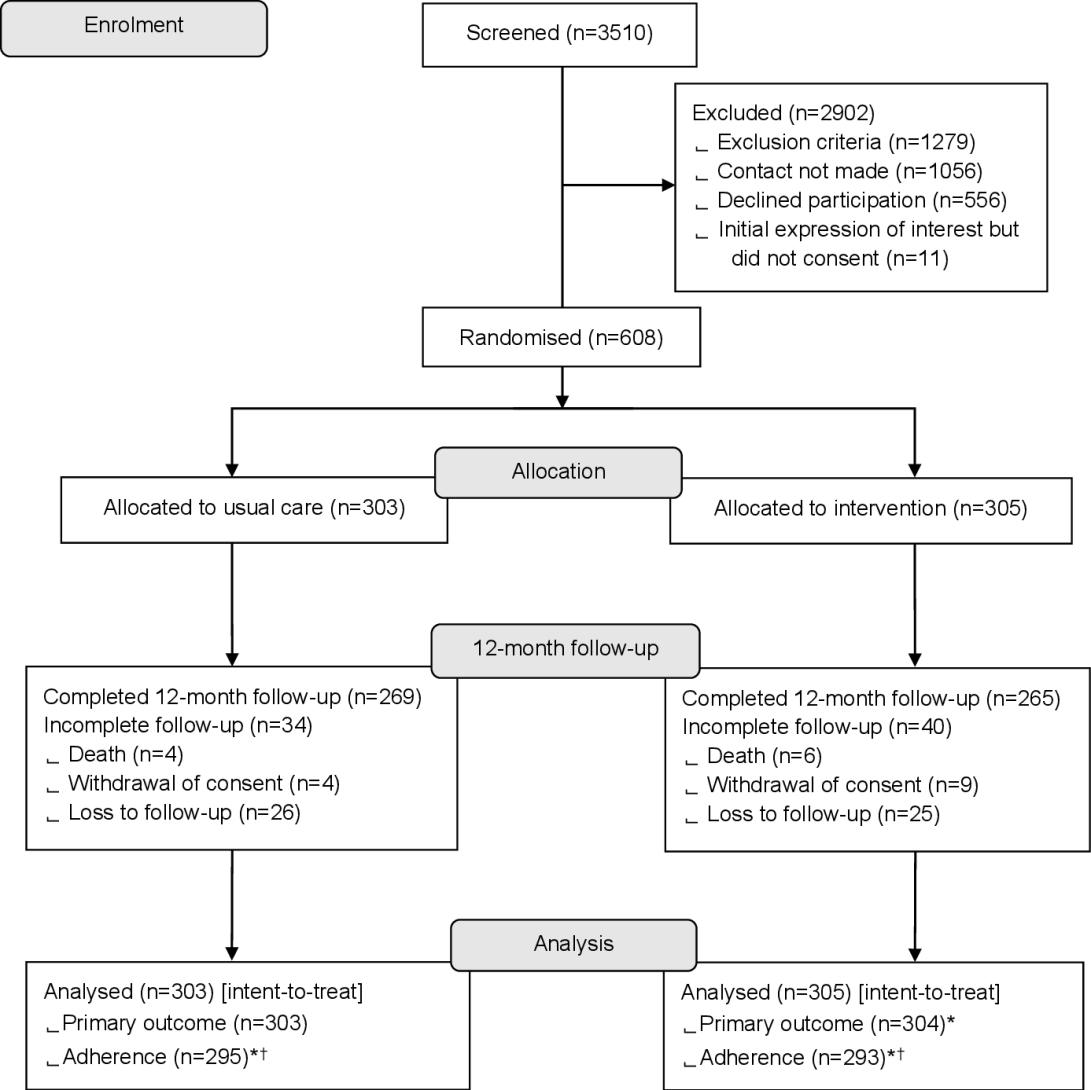
Trial profile. *Exclusions due to missing covariates. ^†^Adherence level to reflect the effect of intervention was calculated using objectively measured effective adherence data from week 3 (ie, from the point of intervention delivery) through to week 52 (ie, the end of the trial), as stated in the ‘Methods’ and ‘Results’. The intervention effect is best reflected by the cumulative adherence level throughout the trial period, similar to the approach of calculating cumulative exacerbation events throughout the trial. Though there were drop-outs during the trial, exacerbation data were available for all participants (expect for a participant who withdrew on the day of randomisation) since exacerbation events prior to drop-out were analysed. In a similar vein, adherence data available prior to the point of drop out were analysed as long as adherence data from week 3 onwards were available. Only 19 participants did not provide any adherence data from week 3 onwards, that is, adherence data were missing for outcome analysis among 19/607 (3%) of participants. Week-by-week breakdown of adherence data completeness is provided in online supplemental table 2.

Between October 2017 and May 2018, 3510 adults with CF were screened, with 608 enrolled and randomised (intervention n=305; usual care n=303) and 556 declined participation (figure 2). Participant recruitment is discussed in appendix C. One participant randomised to intervention withdrew on the day of consent prior to baseline data collection, thus was not included in analyses. The last recruited participant was followed until 30 June 2019, when the trial ended. Baseline demographic and clinical characteristics are in table 2. The intervention group was slightly older, with slightly higher FEV_1_, slightly lower past year intravenous days and slightly higher baseline objectively measured effective adherence (measured 2 weeks post randomisation)—this imbalance is explored in appendix D. Primary outcome data were available for all participants; adherence data were missing for 3.1% (19/607) of participants as explained in figure 2. Week-by-week breakdown of adherence data completeness is in online supplemental table 2.

The median (IQR) number of interventionist sessions per participant was 7.0 (6.0–10.0). The median (IQR) total interventionist delivery time per participant (including contact time and preparation outside of sessions) was 185 (126–263) min. Fidelity of intervention delivery median (IQR) scores were 97.2% (92.3–100.0), 92.6% (87.0–98.1) and 94.4% (91.7–97.2) at the first intervention visits, reviews and phase reviews, respectively (4)).

For the primary outcome, over 12 months, there were 526 pulmonary exacerbations in the usual care arm (adjusted rate 1.77/year, n=303) compared with 482 in the intervention arm (1.63/year, n=304). Incidence rate ratios (95% CI) of the primary analysis (0.96 (0.83 to 1.12); p=0.64), sensitivity analyses (online supplemental table 3) and subgroup analyses (online supplemental figure 1) indicated no significant between-arm difference in exacerbations.

**Figure 3:**
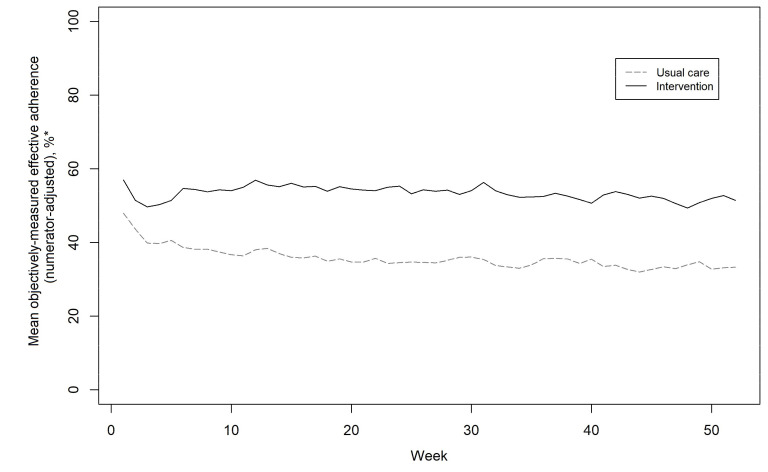
Medication adherence over 12 months, by randomised group (usual care n=295; intervention n=293). *Objectively measured effective adherence (sum of doses taken/sum of doses prescribed) was calculated on a weekly basis, with adjustments made against what may be considered an ideal treatment for effectiveness, as based on the following rules: all participants should receive at least a muco-active agent; and all participants with chronic *Pseudomonas* should receive at least both a mucoactive agent and an antibiotic. Adherence data were aggregated and plotted weekly for the purpose of detecting whether adherence is actually changing to smooth out daily fluctuations that may just be noise, for example, due to weekday versus weekend differences in adherence.^40^

Mean objectively measured effective adherence in weeks 3–52 was 52.9% in the intervention arm versus 34.9% in the usual care arm, with an adjusted mean difference of 9.5 percentage points (95% CI 8.6 to 10.4). Figure 3 shows that adherence declined rapidly at a similar rate in both groups during the first 3 weeks of the trial. The decline among usual care participants continued over the subsequent 12 weeks, then stayed at the level until the end of the trial. In the intervention group, adherence subsequently improved following delivery of the behaviour-change intervention from week 3 onwards (further details in online supplemental appendix D).

Over 12 months, percent predicted FEV_1_ declined by 1.4 (from 58.3±22.6 to 56.9±23.0) among usual care and 0.1 (from 60.7±23.5 to 60.6±24.2) among intervention participants. The adjusted mean differences (95% CI) in per cent predicted FEV_1_ and BMI at 12 months were 1.4 (−0.2 to 3.0) and 0.3 kg/m^2^ (0.1 to 0.6), respectively (table 3).

**Table 3:** Outcomes at 12 months, by randomised treatment group

	Usual care		Intervention		Usual care versus intervention		
	N	Exacerbation rate (no. of exacerbations, person years)	N	Exacerbation rate (no. of exacerbations, person years)	Adjusted difference in means (95% CI)*	Direction of positive effect (possible range)	Standardised effect size
Exacerbations	303	1.77 (526, 297.2)	304	1.63 (482, 294.9)	0.96 (0.83 to 1.12)	Decrease (not applicable)	Not applicable
	**Usual care**		**Intervention**		**Usual care versus intervention**		
	**N**	**Mean±SD**	**N**	**Mean±SD**	**Adjusted difference in means (95% CI)†**	**Direction of positive effect** (**possible range**)	**Standardised effect size**
Objectively measured effective adherence (weekly) – %‡	295	34.9±31.7	293	52.9±31.4	9.5 (8.6 to 10.4)	Increase (0 to 100)	0.29
FEV_1_ % predicted	282	56.9±23.0	274	60.6±24.2	1.4 (−0.2 to 3.0)	Increase (0 to 100)	0.06
Body mass index – kg/m^2^	282	22.6±4.1	273	23.1±4.4	0.3 (0.1 to 0.6)	Increase (not applicable)	0.07
Patient-reported outcomes§							
CFQ-R (quality of life):						Increase (each 0 to 100)	
Physical	274	52.6±30.6	264	55.8±30.2	2.3 (−1.0 to 5.6)		0.08
Emotional	274	66.5±24.7	264	66.6±22.9	0.2 (−2.9 to 3.2)		0.01
Social	274	59.6±20.0	264	60.5±20.0	0.3 (−2.2 to 2.7)		0.01
Eating	274	81.0±23.2	264	84.0±21.5	1.9 (−1.3 to 5.2)		0.09
Body image	274	65.1±29.3	264	67.2±27.3	1.7 (−1.4 to 4.8)		0.06
Treatment burden	274	51.5±19.7	265	56.6±19.5	3.9 (1.2 to 6.7)		0.20
Respiratory	271	56.6±21.9	263	58.0±22.5	0.7 (−2.4 to 3.8)		0.03
Digestion	272	80.2±21.6	263	80.4±19.4	1.1 (−1.7 to 3.9)		0.05
BMQ-Specific (beliefs about medication):							
Concerns	271	2.1±0.5	271	2.0±0.5	−0.2 (−0.2 to –0.1)	Decrease (1 to 5)	0.29
Necessities	271	3.5±0.7	271	3.7±0.8	0.1 (0.0 to 0.2)	Increase (1 to 5)	0.18
SRBAI (habit strength for using nebuliser)	271	11.7±4.9	261	12.9±4.9	1.2 (0.5 to 1.8)	Increase (4 to 20)	0.24
Perceptions of treatment adherence (three-item scale)	245	9.9±3.6	237	10.8±3.3	0.7 (0.2 to 1.2)	Increase (3 to 15)	0.20
Effort of nebuliser treatments (one item)	270	3.0±1.2	260	3.3±1.3	0.3 (0.1 to 0.5)	Increase (1 to 5)	0.22
Subjective adherence question – % (self-report estimate of adherence)	267	65.6±32.8	258	68.6±31.3	1.9 (−2.8 to 6.6)	Increase (0% to 100%)	0.06
CHAOS-6 (life chaos or routine)	272	9.6±3.2	263	9.4±3.4	−0.2 (−0.6 to 0.3)	Decrease (0 to 24)	0.05
PAM-13 (health style assessment)	274	64.9±13.0	265	68.1±15.6	3.4 (1.3 to 5.4)	Increase (0 to 100)	0.23
EQ-5D-5L (generic health status)	272	0.81±0.18	264	0.84±0.15	0.01 (−0.01 to 0.04)	Increase (−0.224 to 1)	0.09
Patient-reported outcomes – safety measures§							
PHQ-8 (depression)	272	6.4±5.0	262	6.3±5.6	−0.1 (−0.8 to 0.7)	Decrease (0 to 24)	0.01
GAD-7 (anxiety)	273	4.5±4.8	262	4.9±5.3	0.3 (−0.4 to 1.0)	Decrease (0 to 21)	0.05

Full details and references for all patient-reported outcomes are available in the SAP (provided in online supplemental material).

*Exacerbations analysis adjusted for centre and past year intravenous days.

†All other analyses adjusted for past year intravenous days, centre and outcome measure at baseline.

‡Weekly objectively measured effective adherence (sum of doses taken/sum of doses prescribed) averaged over weeks 3–52 postrandomisation.

§All patient-reported outcomes based on points, unless otherwise stated.

BMQ, Beliefs About Medicines Questionnaire; CFQ-R, CF Questionnaire-Revised; CHAOS-6, Confusion, Hubbub and Order 6-item Scale; EQ-5D-5L, EuroQol 5-dimension and 5-level; GAD-7, Generalised Anxiety Disorder seven-item scale; PAM-13, Patient Activation 13-item Measure; PHQ-8, Patient Health Questionnaire eight-item depression scale; SAP, statistical analysis plan; SRBAI, Self-Report Behavioural Automaticity Index.

Of the eight CFQ-R subscales, seven showed no between-group difference, but there was lower perceived CF treatment burden (3.9 (1.2, 6.7) points) in the intervention arm. Of the other 11 patient-reported outcomes at 12 months (including two safety measures; table 3), six showed differences for intervention versus usual care (adjusted difference in means (95% CI)), with increases in beliefs about medication necessities (0.1 (0.0 to 0.2) on BMQ-Specific necessities), habit strength for using nebuliser (1.2 (0.5 to 1.8) on SRBAI), perceptions of treatment adherence (0.7 (0.2 to 1.2) on three-item scale) and patient activation (3.4 (1.3 to 5.4) on PAM-13), and decreases in concerns about treatment (0.2 (0.1, 0.2) on BMQ-Specific concerns) and perceived effort of nebuliser treatments (0.3 (0.1 to 0.5) for one item). Other outcomes, including the safety measures of depression (−0.1 points (−0.8 to 0.7) on PHQ-8) and anxiety (0.3 points (−0.4 to 1.0) on GAD-7), showed no between-group difference.

No intervention-related serious adverse events were reported (online supplemental table 4). Sixty-four serious adverse events (21 in expected categories due to disease or treatment) in 43 usual care participants were recorded and 71 (28 expected) in 56 intervention participants.

## Discussion

In this randomised, usual care-controlled trial, we investigated the effectiveness of a self-management intervention designed to reduce pulmonary exacerbation rates among adults with CF, by supporting their adherence to inhaled mucoactive agents and antibiotics. Over 12 months, a significant difference in pulmonary exacerbations or FEV_1_ was not detected. However, compared with usual care, the intervention achieved higher objectively measured effective adherence, higher BMI and lower perceived CF treatment burden.

It is possible that the primary outcome was not achieved due to insufficient between-group difference in adherence. It is also possible that using exacerbation as an endpoint was problematic. Exacerbation is a robust, sensitive outcome for blinded efficacy drug trials,^21^ but an unblinded, pragmatic evaluation of self-management support presents challenges to its use. There is a discretionary element to the use of intravenous antibiotics as rescue therapy; previous studies suggest only around 50% of events meeting 3/4 Rabin exacerbation criteria or acute 10% decline in FEV_1_ receive additional antibiotics.^26^ Increasing a person’s adherence to treatment may improve their acceptance of intravenous antibiotics,^27^ and more intense monitoring can detect more exacerbations.^28^ It is possible that increased clinician contact time in the intervention group created differential surveillance that biased the exacerbation rate towards unity (ascertainment bias). In UK practice, intravenous antibiotics will always be started by the CF care team, whereas oral antibiotics can be started in the community and may be much more susceptible to differential surveillance. To avoid this bias, oral courses were not collected. As a consequence, it is possible that improvement in milder exacerbations may have been missed. Adding a standardised criteria, for example, the Fuchs criteria, as part of the definition allowed exacerbation measurement across different centres to be comparable. However, recent work has suggested that this may result in reduced sensitivity.^29^

The graph for objectively measured effective adherence (figure 3) has several features that merit discussion (further details in appendix D). The pragmatic trial design of providing data-logging nebulisers and revealing trial allocation to participants on day 1, then measuring baseline adherence in the first 2 weeks of the trial creates complexity. The baseline between-group difference in objectively measured effective adherence was 8.6% in favour of the intervention group (54.1%±33.0% vs 45.5±34.1%), with the intervention group being older yet having higher FEV_1_ and lower IV days in the 12 months prior to study entry. Over the 49 weeks of the intervention, there was a between-group difference in objectively measured effective adherence of 18.0% in favour of the intervention group (52.9%±31.4% vs 34.9±31.7%), with an adjusted mean difference of 9.5% (95% CI 8.6% to 10.4%) taking into account the baseline adherence. Analysis comparing the adherence response stratified by baseline adherence (appendix D (figure D2)) shows that a significant between-group difference in adherence emerged at all levels of baseline adherence, emphasising that there was genuine divergence in adherence between the intervention and usual care independent of baseline adherence. It can be seen (figure 3) that in both groups there was an initial rapid decline in adherence. Among usual care participants not receiving any intervention, this decline continued over the next 12 weeks to around 35% and stayed at this level until the end of the trial. Real-world objective inhaled therapy monitoring has demonstrated similar levels of adherence among adults with CF not receiving intervention.^8 9^ In the intervention group, the initial rate of decline was similar to usual care until the behavioural change intervention started from week 3 onwards and adherence subsequently improved. Given the rapid initial decline of both groups over the first 3 weeks of the study, it seems possible that the adherence at study entry was a short-term manifestation of device novelty^30^ and white coat adherence.^31^

Since people with low adherence may find adherence data threatening,^32^ it is important to note that the differential adherence in the intervention arm was achieved without increasing anxiety. The intervention also achieved increase in necessity and reduction in concerns for treatment taking, consistent with literature highlighting that self-management of LTCs may be improved by addressing treatment beliefs.^17^ Policy makers who emphasise the importance of patient activation in LTCs^33^ can be reassured that the intervention significantly increased knowledge, skills and confidence (patient activation). The intervention achieved clinically important improvements in perceived treatment burden,^34^ which was identified as the number one research priority by the CF community.^12^ That total nebuliser use should increase while the perceived burden and effort of nebuliser treatment decrease may relate to a moderating role for habit.^18 19^ Literature in LTCs emphasises that sustained adherence is generally more strongly associated with habit than reflective motivation, which is more effortful.^35^

In considering the effective components of the intervention, data feedback is an obvious candidate. However, participants consulted data infrequently outside of supervised sessions. A qualitative analysis undertaken as part of the pilot work reported the value of the range of behaviour change techniques used in the intervention as well as the importance of building a relationship with the interventionist.^36^ It is unlikely that unsupported feedback alone is sufficient to explain the reduced treatment burden, the improved necessity and concerns for treatment or the increased habit strength.

A strength of the trial is the automatic capture of objective adherence with data-logging nebulisers that record every dose taken. Online supplemental table 2 demonstrates similar levels of week-by-week data completeness for both groups. Robust adherence data allowed us to demonstrate a sustained adherence difference for 12 months, which is the first for inhaled medications in any LTC. Sustained objectively measured adherence benefits for behavioural interventions in other LTCs are limited to two studies, both for oral medications among older adults in the hypertension and post-transplantation settings.^37 38^ CFHealthHub as a multi-component self-management intervention has now been established as a digital learning health system (ISRCTN14464661) in >50% of UK adult CF centres. Limitations of the trial include the delivery of both behavioural change and research procedures by interventionists, a period of server downtime that affected intervention delivery, the fact that the trial powered for exacerbation was not designed to detect the observed point estimate in FEV_1_ and the recruitment of a convenience sample whereby a third of the participants had objectively measured effective adherence levels >75%. The vulnerability of adherence studies to differential inclusion of more engaged patients is likely to reduce both the impact of the adherence intervention on studied behaviour and reduce the impact on health outcomes.^39^ This may mean that the positive behavioural findings observed in this study are particularly noteworthy. The direction of bias and implications of these limitations are further discussed in appendix E.

In this randomised controlled trial, an intervention for adults with CF that combines measurement of objective adherence to prescribed medication using data-logging nebulisers, a digital platform and manualised behavioural-change sessions delivered by trained clinical interventionists did not significantly affect pulmonary exacerbations and FEV_1_ but did result in higher objectively measured effective adherence, higher BMI and lower perceived CF treatment burden versus usual care, without increasing anxiety. This is the first iteration of a self-management intervention that may have the potential to be improved by continual iteration in a digital learning health system. Analogous to the overwhelming success in the CF drug pipeline of building on early signals with ongoing developments and trials, we plan to continue iterating and evaluating the CFHealthHub-based intervention by building on signals we have observed to further improve the intervention. Given that adherence is low in LTCs and that prescribed medications only work if taken appropriately, focusing on further evaluation of adherence interventions is important.

## Data Availability

Data are available on reasonable request. Requests for patient level data and statistical code should be made to the corresponding author and will be considered by members of the original trial management group, including the chief investigators and members of clinical trials research unit, who will release data on a case-by-case basis. Data will be shared following the principles for sharing patient level data as described by Tudur Smith C, *et al BMC Medicine* 2015;13:298 (https://doi.org/10.1186/s12916-015-0532-z). The data will not contain any direct identifiers, and we will minimise indirect identifiers and remove free-text data to minimise the risk of identification.
