# Supplementary material for: Self-management intervention to reduce pulmonary exacerbations by supporting treatment adherence in adults with cystic fibrosis: a randomised controlled trial
Source: Thorax. 2021 Sep 23;77(5):461–9. doi: 10.1136/thoraxjnl-2021-217594 (PMC9016257; doi:10.1136/thoraxjnl-2021-217594)
Supplement: Supplementary data [file thoraxjnl-2021-217594supp001.pdf]

SUPPLEMENTARY MATERIAL

Self-management intervention to reduce pulmonary exacerbations by supporting treatment adherence in adults with cystic fibrosis: a randomised controlled trial

Martin J Wildman,[1,2] Alicia O'Cathain,[2] Chin Maguire,[3] Madelynne A Arden,[4] Marlene Hutchings,[1] Judy Bradley,[5] Stephen J Walters,[2] Pauline Whelan,[6] John Ainsworth,[6] Iain Buchan,[6,7] Laura Mandefield,[2] Laura Sutton,[2] Paul Tappenden,[2] Rachel A Elliott,[8] Z Hui Hoo,[1,2] Sarah J Drabble,[2] Daniel Beever,[3]
for the CFHealthHub Study Team

CONTENTS

Table S1 Cluster sample size calculations for comparison of continuous data – two independent groups

Table S2 Objectively-measured effective adherence weekly summaries (complete case), by randomised treatment group

Table S3 Primary outcome sensitivity analyses over 12 months, by randomised treatment group

Figure S1 Primary outcome subgroup analysis according to baseline objectively-measured effective adherence level

Table S4 Adverse events and serious adverse events over 12 months, by randomised treatment group

APPENDIX A Description of the CFHealthHub intervention

APPENDIX B The choice of adherence measure

APPENDIX C Participant recruitment

APPENDIX D Between-group imbalance and “baseline adherence”

APPENDIX E Other limitations of the trial

Table S1 Cluster sample size calculations for comparison of continuous data – two independent groups

PLEASE ENTER Mean1, Mean2, SD, IntraCluster Correlation Coefficient Average Cluster Size

Significance Level	POWER	$Mean_1$	$Mean_2$	SD	Mean Difference	Standardised Effect Size	Intra Cluster Correlation	Mean Cluster Size	iRCT † No. in each group	Total Sample Size	Total Sample Size Drop out 15%	Design Effect Inflation Factor	cRCT ‡ No. in each group	Total Sample Size	Total Sample Size Drop out 15%	
**Exacerbations Outcome**																
5%	90%	3	2	3	1	0.33	0.000	25	191	382	450	1.00	191	382	450	**Original sample calculation with no clustering**
5%	90%	3	2	3	1	0.33	0.010	25	191	382	450	1.24	237	474	558	Original sample calculation with clustering
5%	90%	3	2	3	1	0.33	0.035	70	191	382	450	3.42	653	1306	1538	Cluster RCT calculation
5%	90%	3	2	3	1	0.33	0.010	25	191	382	450	1.24	237	474	558	
5%	90%	3	2.1	3	0.9	0.30	0.010	25	235	470	554	1.24	292	584	688	***Proposed sample size with allowance for contamination and clustering by health team***
**FEV_1 outcome**	**80% power**															
5%	80%	1	0	25	1	0.04	0.010	25	9813	19626	23090	1.24	12169	24338	28634	
5%	80%	2	0	25	2	0.08	0.010	25	2454	4908	5776	1.24	3043	6086	7160	
5%	80%	3	0	25	3	0.12	0.010	25	1092	2184	2570	1.24	1355	2710	3190	
5%	80%	4	0	25	4	0.16	0.010	25	615	1230	1448	1.24	763	1526	1796	
5%	80%	5	0	25	5	0.20	0.010	25	394	788	928	1.24	489	978	1152	
5%	80%	6	0	25	6	0.24	0.010	25	274	548	646	1.24	340	680	800	
5%	80%	7	0	25	7	0.28	0.010	25	202	404	476	1.24	251	502	592	
5%	80%	8	0	25	8	0.32	0.010	25	155	310	366	1.24	193	386	456	
5%	80%	9	0	25	9	0.36	0.010	25	123	246	290	1.24	153	306	360	
5%	80%	10	0	25	10	0.40	0.010	25	100	200	236	1.24	124	248	292	
**FEV_1 outcome power with N=688 randomised**																
5%	7%	1	0	25	1	0.04	0.000	0	292	584	688	1.00	292	584	688	Sample size fixed at N~ 688 randomised
5%	16%	2	0	25	2	0.08	0.000	0	292	584	688	1.00	292	584	688	Size of difference in FEV_1 able to detect
5%	30%	3	0	25	3	0.12	0.000	0	292	584	688	1.00	292	584	688	
5%	49%	4	0	25	4	0.16	0.000	0	292	584	688	1.00	292	584	688	
5%	67%	5	0	25	5	0.20	0.000	0	292	584	688	1.00	292	584	688	
5%	76%	5.5	0	25	5.5	0.22	0.000	0	292	584	688	1.00	292	584	688	
5%	83%	6	0	25	6	0.24	0.000	0	292	584	688	1.00	292	584	688	
5%	88%	6.5	0	25	6.5	0.26	0.000	0	292	584	688	1.00	292	584	688	
5%	92%	7	0	25	7	0.28	0.000	0	292	584	688	1.00	292	584	688	
5%	97%	8	0	25	8	0.32	0.000	0	292	584	688	1.00	292	584	688	

*Power calculations demonstrated greater efficiency of pulmonary exacerbations versus FEV_1, thereby influencing the choice of pulmonary exacerbations as the primary outcome.

†Randomisation in the trial was on an individual rather than cluster basis because our power calculation indicated a requirement for 1,400 adults across 20 centres for cluster randomisation, which would not be feasible. Although contamination is a risk with individual randomisation, this can often be overcome by increasing the sample size. In most cases, individual randomisation accounting for contamination requires a smaller sample size than cluster randomisation.[1]

FEV_1, forced expiratory volume in one second; RCT, randomised controlled trial (c, cluster; i, individual); SD, standard deviation.

Reference

1. Torgerson DJ. Contamination in trials: is cluster randomisation the answer? *BMJ* 2001;322:355–7.

Table S2 Objectively-measured effective adherence weekly summaries (complete case), by randomised treatment group

Week	Usual care		Intervention	
	**N**	**Mean (SD)**	**N**	**Mean (SD)**
1	289	48.0 (35.0)	290	57.0 (34.2)
2	295	43.7 (35.1)	293	51.4 (34.6)
3	298	39.9 (34.8)	295	49.7 (34.3)
4	297	39.7 (35.4)	297	50.3 (35.1)
5	293	40.5 (34.9)	298	51.4 (34.9)
6	291	38.6 (34.5)	299	54.7 (34.7)
7	291	38.2 (35.1)	298	54.4 (35.2)
8	292	38.1 (35.9)	298	53.8 (36.1)
9	292	37.4 (35.3)	297	54.3 (35.0)
10	291	36.6 (34.7)	297	54.0 (35.9)
11	290	36.4 (34.8)	297	54.9 (35.6)
12	290	38.0 (34.9)	297	56.9 (35.7)
13	290	38.4 (35.6)	296	55.6 (36.4)
14	290	37.0 (35.2)	294	55.1 (36.9)
15	289	36.0 (34.9)	293	56.1 (36.8)
16	286	35.8 (34.8)	293	55.1 (36.3)
17	286	36.3 (34.4)	293	55.2 (35.7)
18	285	34.9 (34.6)	293	53.9 (35.6)
19	285	35.5 (34.7)	293	55.2 (35.1)
20	285	34.6 (35.1)	293	54.5 (36.0)
21	283	34.7 (36.5)	292	54.2 (37.1)
22	283	35.7 (36.6)	292	54.1 (36.3)
23	283	34.2 (35.7)	291	55.0 (36.4)
24	282	34.5 (34.8)	290	55.3 (35.8)
25	282	34.7 (34.0)	290	53.2 (35.9)
26	281	34.6 (33.8)	290	54.3 (36.0)
27	281	34.4 (34.5)	288	53.9 (36.6)
28	279	35.2 (35.9)	288	54.2 (35.7)
29	280	36.0 (35.8)	287	53.1 (36.0)
30	276	36.0 (35.9)	287	54.1 (36.4)
31	275	35.4 (35.5)	285	56.3 (36.5)
32	274	33.7 (34.0)	285	54.1 (36.7)
33	274	33.3 (34.5)	284	53.0 (37.2)
34	274	33.0 (33.5)	283	52.3 (36.8)
35	273	33.9 (34.5)	282	52.3 (36.7)
36	273	35.6 (35.1)	281	52.5 (36.4)

37	272	35.6 (34.3)	279	53.3 (35.6)
38	272	35.5 (35.1)	279	52.6 (36.1)
39	272	34.2 (34.8)	279	51.6 (37.2)
40	272	35.5 (35.4)	276	50.6 (37.4)
41	272	33.4 (34.2)	275	52.9 (35.9)
42	272	33.8 (33.9)	274	53.8 (36.2)
43	272	32.7 (35.1)	274	53.1 (36.4)
44	271	32.0 (34.4)	272	52.0 (37.3)
45	271	32.7 (34.9)	272	52.6 (36.6)
46	269	33.3 (34.8)	272	52.0 (36.4)
47	269	32.9 (34.5)	271	50.6 (37.4)
48	269	33.9 (35.7)	271	49.3 (37.1)
49	269	34.7 (35.6)	269	50.8 (36.6)
50	268	32.8 (35.8)	269	52.0 (36.0)
51	267	33.1 (35.3)	269	52.7 (35.9)
52	266	33.2 (35.0)	268	51.4 (36.1)

SD, standard deviation.

Table S3. Primary outcome sensitivity analyses over 12 months, by randomised treatment group

Sensitivity analysis*	Usual care				Intervention				Incidence rate ratio (95% CI)	P value
	N	Exacerbations	Person-years	Exacerbation rate	N	Exacerbations	Person-years	Exacerbation rate		
Main – adjusted	303	526	297.2	1.77	304	482	294.9	1.63	0.96 (0.83, 1.12)	0.638
Main – unadjusted	303	526	297.2	1.77	304	482	294.9	1.63	0.92 (0.77, 1.11)	0.387
All exacerbations†	303	558	297.2	1.88	304	504	294.9	1.71	0.95 (0.82, 1.10)	0.511
MICE	303	–	–	–	304	–	–	–	0.98 (0.84, 1.15)	0.821
Best case imputation	303	526	297.2	1.77	304	482	301.9	1.60	0.94 (0.81, 1.10)	0.444

Model definitions:

Main – adjusted for stratification factors (centre and past-year IV days)

Main – unadjusted for any covariates except duration of post-consent follow-up

All exacerbations – main model including additional exacerbations meeting Fuchs' criteria but not treated with parenteral antibiotics

MICE – missing count data imputed (where missingness not due to death) using randomization group, site, previous year's IV days, age, gender, FEV_1 % predicted, *Pseudomonas* status, and exacerbation count

Best case imputation – missing intervention arm follow-up time imputed (where missingness not due to death) assuming no further exacerbations

Recurrent event survival – extension of proportional hazards time-to-event model allowing for repeat events (exacerbations) with no assumption of constant event rate

*Recurrent event survival was also calculated: hazard ratio 0.95 (95% CI 0.80, 1.13; p=0.567).

†The difference between 'all exacerbations' and 'main – unadjusted' is the number of IV antibiotic courses that were offered by clinicians but declined by participants. The IV-declined rate was 32/558 (5.7%) for the usual care arm and 22/504 (4.4%) for the intervention arm. These values are far lower than the IV-declined rate observed in the general CF population of around 20%,[1] which provides evidence that the recruited participants may not be representative of the general CF population.

BMI, body mass index; CF, cystic fibrosis; CI, confidence interval; EQ-5D-5L, EuroQol 5-dimension and 5-level;

FEV_1, forced expiratory volume in one second; IV, intravenous; MICE, multiple imputation using chained equations.

Reference

1. Hoo ZH, Bramley NR, Curley R, *et al.* Intravenous antibiotic use and exacerbation events in an adult cystic fibrosis centre: a prospective observational study. *Respir Med* 2019;154:109–15.

Figure S1 Primary outcome subgroup analysis according to baseline objectively-measured effective adherence level. CI, confidence interval; IRR, incidence rate ratio.

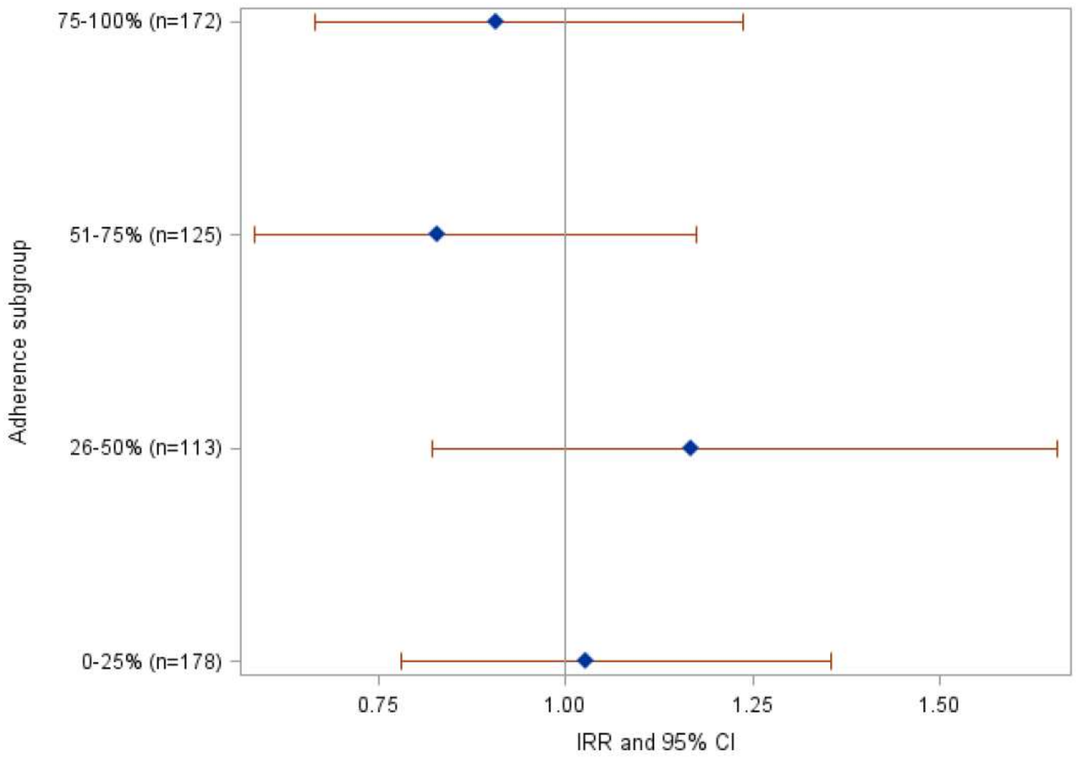

Statistical significance was not observed in any of the subgroups by baseline objectively-measured effective adherence. “Pulmonary exacerbation” was defined as the administration of intravenous (IV) antibiotics for any of the 12 Fuchs’ symptoms/signs. As discussed in the main manuscript, there is a discretionary element to the use of IV antibiotics as rescue therapy to treat exacerbations. Increasing a person’s adherence to inhaled therapies may also improve their engagement with other treatments, including improving their acceptance of IV rescue antibiotics. It is possible that the exacerbation rate appeared to have somewhat increased in those with baseline adherence 26–50% because this is the subgroup with greatest improvement in adherence, potentially leading to the greatest impact from ascertainment bias (see appendix D [figure D2] for the subgroup analysis of adherence). It should be noted that interactions between exacerbations and adherence are complex, including for example the impact of engagement on IV acceptance, such that interpretation should be made with caution.

Table S4 Adverse events and serious adverse events over 12 months, by randomised treatment group

	**Usual Care (N=303)**	**Intervention (N=305)**
**All AE**		
Number of AE, overall – n (% of all AE)	301 (46.9)	341 (53.1)
Number of participants experiencing ≥1 AE – n (% of participants in treatment arm)	125 (41.3)	139 (45.6)
Number of AE, by category – n (% of AE in treatment arm)		
Expected*	242 (80.4)	263 (77.1)
Other	58 (19.3)	73 (21.4)
**Serious AE*†**		
Number of serious AE, overall – n (% of all serious AE)	64 (47.4)	71 (52.6)
Number of participants experiencing ≥1 serious AE – n (% of participants in treatment arm)	43 (14.2)	56 (18.4)
Number of serious AE, by category – n (% of serious AE in treatment arm)		
Expected*	21 (32.8)	28 (39.4)
Other	41 (64.1)	42 (59.2)
Unknown	2 (3.1)	1 (1.4)

*Certain AE common to CF and associated medications were categorised as expected. Examples of expected AE include acute FEV_1 drop >15% after first dose of medication, increased productive cough and nasal congestion. The full list of expected AE is provided in section 12.3.3 of the protocol (available as supplementary material).

†There were no serious AE deemed related to the intervention (non-serious AE were not assessed for relatedness).

AE, adverse event; CF, cystic fibrosis; FEV_1, forced expiratory volume in one second.

APPENDIX A Description of the CFHealthHub intervention

Aim

The CFHealthHub intervention aims to support adults with cystic fibrosis (CF) to increase and maintain their adherence to prescribed nebulised medication in order to reduce exacerbations and improve or prevent decline in lung function.

Rationale

The CFHealthHub intervention is underpinned by the Capability Opportunity Motivation-Behaviour (COM-B) model.[1] It has been developed using the Behaviour Change Wheel approach alongside a person-based approach to intervention development. This process is described in detail elsewhere[2] but broadly consisted of the following stages:

- Identification of barriers and facilitators for nebuliser adherence using the Theoretical Domains Framework
- Identification of appropriate intervention functions and behaviour change techniques to address barriers identified
- Iterative development of the CFHealthHub intervention with patients, using feedback from interviews and 'think aloud' to refine the intervention
- Creation of an intervention manual and training programme for interventionists
- Pilot and feasibility trial including a process evaluation which was used to further refine the intervention, manual and training process

Conceptual framework and theory

The conceptual framework that describes the intervention is provided in figure A1. Consistent with the COM-B model, the framework considers issues of capability, opportunity and motivation, all of which must be present in order for repetition of the behaviour (i.e. medication adherence) to occur. Initially we anticipate that repetition will require effortful self-regulation, but with repetition and strategies to promote habit formation we aim for the behaviour to become more automatic.

Figure A1 Sustained behaviour conceptual framework.

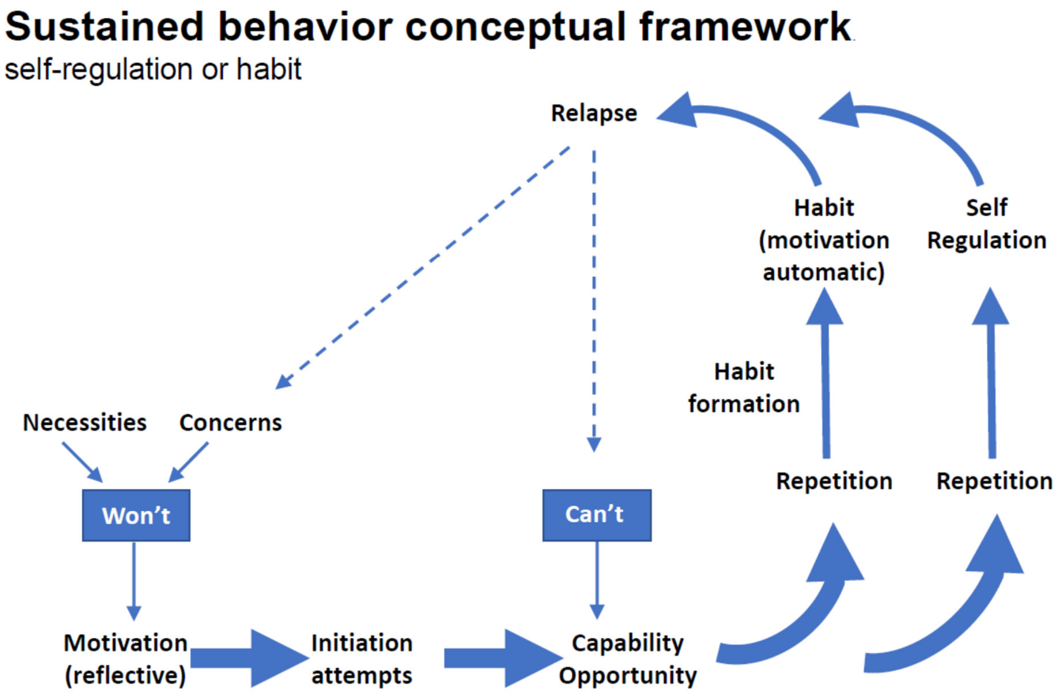

The intervention addresses a range of different barriers and is tailored to meet the specific needs of the person. The intervention draws on key theories in order to address different parts of the proposed process: Social Cognitive Theory,[3] Control Theory,[4] and Habit Theory,[5] as follows:

- Social Cognitive Theory (SCT)[3] proposes that behaviour is influenced by two core constructs: i) perceived self-efficacy, i.e. an individual's beliefs in their capability to adhere to treatment; and ii) outcome expectancies, i.e. an individual's beliefs about the likely consequences of their actions. Self-efficacy can be enhanced through: i) mastery; ii) vicarious experiences, where a role model, similar to the individual successfully achieves behavioural change in a similar situation; or iii) verbal persuasion. Outcome expectancies include beliefs about the positive and negative and short- and long-term consequences of adherence, and in this context include perceived necessities and concerns.[6] According to SCT, outcome expectancies may result in intentions to change one's behaviour. Self-efficacy then influences the translation of that intention into action through the pursuit of goals.

- Control Theory[4] explains the processes of self-regulation. When a behavioural standard or goal has been set, an individual directs their attention through monitoring behaviour to the discrepancy between their current behaviour and their goal. They then use this feedback to regulate their behaviour to meet their goal through action control. This in the context of adherence, once an adherence goal is set, self-monitoring of treatment-taking provides the feedback to prompt action to enable self-regulation of behaviour.
- A habit is where a behaviour is prompted automatically by a situational cue. Habits are created due to the repetition of a behaviour in a specific context[7] which, over time results in a learned cue-behaviour association.[8] In the context of adherence, the repeated taking of treatment in a specific context or in the presence of a specific cue should over time result in the formation of a habit. Habits are particularly advantageous because theory predicts that, once formed, they do not rely on motivational processes and therefore should persist even if motivation wanes.[9] They may therefore play a particularly important role in the promotion of long-term maintenance of behaviour,[10] in this case adherence which is a key aim of the programme.

Materials

The CFHealthHub intervention includes a range of materials as follows:

1. eFlow Technology nebulisers with eTrack data-logging Controllers (PARI Pharma GmbH, Starnberg, Germany)
2. 2net Hub (Capsule Technologies, San Diego, USA)
3. Research procedures manual
4. CFHealthHub web platform
5. CFHealthHub app (available for Apple and Android devices)
6. COM-B Beliefs about Medicines Questionnaire (COM-BMQ) screening tool
7. CFHealthHub Participant manual
8. CFHealthHub Interventionist manual including worksheets for intervention delivery
9. Training slides, and online resources (via Blackboard virtual learning environment [VLE]) for interventionist training
10. Fidelity scoring sheets

Intervention providers

Intervention providers were recruited from each site. The majority of sites recruited individuals who were already members of the multi-disciplinary teams working in CF at that site. Other sites recruited from other parts of the hospital or recruited externally.

Thus, interventionists had a range of backgrounds including:

- Physiotherapists working in CF or other respiratory conditions
- Nurses working in CF
- Psychologists
- Pharmacists
- Dieticians

Procedure

Interventionist training, assessment and support

Interventionists received training in how to deliver the intervention in a variety of ways:

1. Training in use of equipment

Interventionists received training in how to use the eTrack nebuliser and 2net Hub, how to pair the devices, and how to register a new participant onto the CFHealthHub platform and PARI Track system, as part of their research procedures training. This was delivered face-to-face by the study manager and PARI, and supported with a research procedures manual and ad-hoc telephone support throughout the trial.

2. Training in delivery of CFHealthHub intervention

Interventionists received training in how to use the CFHealthHub web platform and how the deliver the CFHealthHub intervention. Training was delivered over a 2-day face-to-face training session, followed by a schedule of online training to be completed over the equivalent of 4 days hosted by the Blackboard VLE. Training consisted of presentations with exercises in small groups or pairs, supported use of CFHealthHub, role play delivery of the intervention and discussion. A training version of the CFHealthHub platform was provided for use during training that included dummy data. Interventionists were paired to form buddies for support and additional role play during the online part of the training.

3. Competency assessment

Interventionists undertook two competency assessments during the training period:

i. Theory test, which assessed understanding of the content of the CFHealthHub web platform content and data. This test was delivered through an online survey on the VLE and consisted of multiple choice and short answer questions. The answers were marked according to a pre-determined marking schedule. Interventionists passed if they received a mark of ≥80%. Individual feedback was provided on the answers given; where the first test was failed, additional tutorial support was provided and the test retaken until passed.
ii. Practical test, which assessed delivery of the first intervention visit of the CFHealthHub intervention. This was assessed through an audio-recorded role play. The part of the participant was played by a member of the study team and the interventionist role-played their part. The intervention delivery was assessed using a competency assessment sheet which consisted of sections on preparation, delivery of intervention components, and the quality of delivery. Two members of the training team looked at the completed worksheet for the session and listened to the accompanying audio-recording. They then discussed the marks and agreed marks where there were any differences. Agreed marks for each section were averaged and the pass mark was 90%. Interventionists received individual feedback on their performance and tutorial support where they had failed. The test was retaken until passed.

Competencies to deliver a review visit and a phase review visit were assessed by listening to the first audio-recorded visit of that kind for each interventionist. Two members of the training team looked at the completed worksheet for the session and listened to the accompanying audio-recording. They then discussed and agreed marks. Agreed marks for each section were averaged and the pass mark was 90%. Interventionists received individual feedback on their performance and tutorial support where they had failed. The next audio-recorded visit of that kind was assessed where the assessment was failed.

4. Ongoing support

Ongoing support for interventionists was delivered via a weekly teleconference, email and telephone support with the training team, technical support via telephone and email. The weekly teleconference provided a space where interventionists could discuss problems, successes and case studies (anonymised), to aid group learning. Individuals could also access members of the team individually and individual interventionists were targeted with support where they had failed their earlier competency assessment or where there were any problems identified.

Intervention schedule of delivery

The intervention schedule of delivery is described in figure A2. The content of each kind of intervention session is described below. Within this schedule there are a number of different paths that were determined during delivery.

Consent visit and set-up

All participants receive their eTrack nebuliser and 2net Hub at the consent visit. They also complete the COM-BMQ screening tool at this visit. An account is created on CFHealthHub into which is added the current prescription data for the participant and the data from the COM-BMQ screening tool. The consent visit takes place ≥4 weeks prior to the first intervention visit. During this time adherence data is transmitted automatically from the eTrack nebuliser via the 2net Hub, which is plugged into their home, to the CFHealthHub platform. Figure A3 shows this process.

Intervention sessions received by all participants

All participants receive their first intervention visit ≥4 weeks following consent (so that the consultation is based on ≥4 weeks' worth of objectively-measured adherence data). This visit is always done face-to-face although can be in a variety of locations, including hospital (in-patient), clinic or home. All participants then receive an intermediate review phone call one week later. Subsequent visits depend on their objectively-measured effective adherence level. Participants with an adherence level of ≥80% follow the 'Very high adherence' pathway while those with adherence level of <80% follow the normal pathway.

Figure A2 Schedule of intervention delivery. IV, intravenous antibiotics.

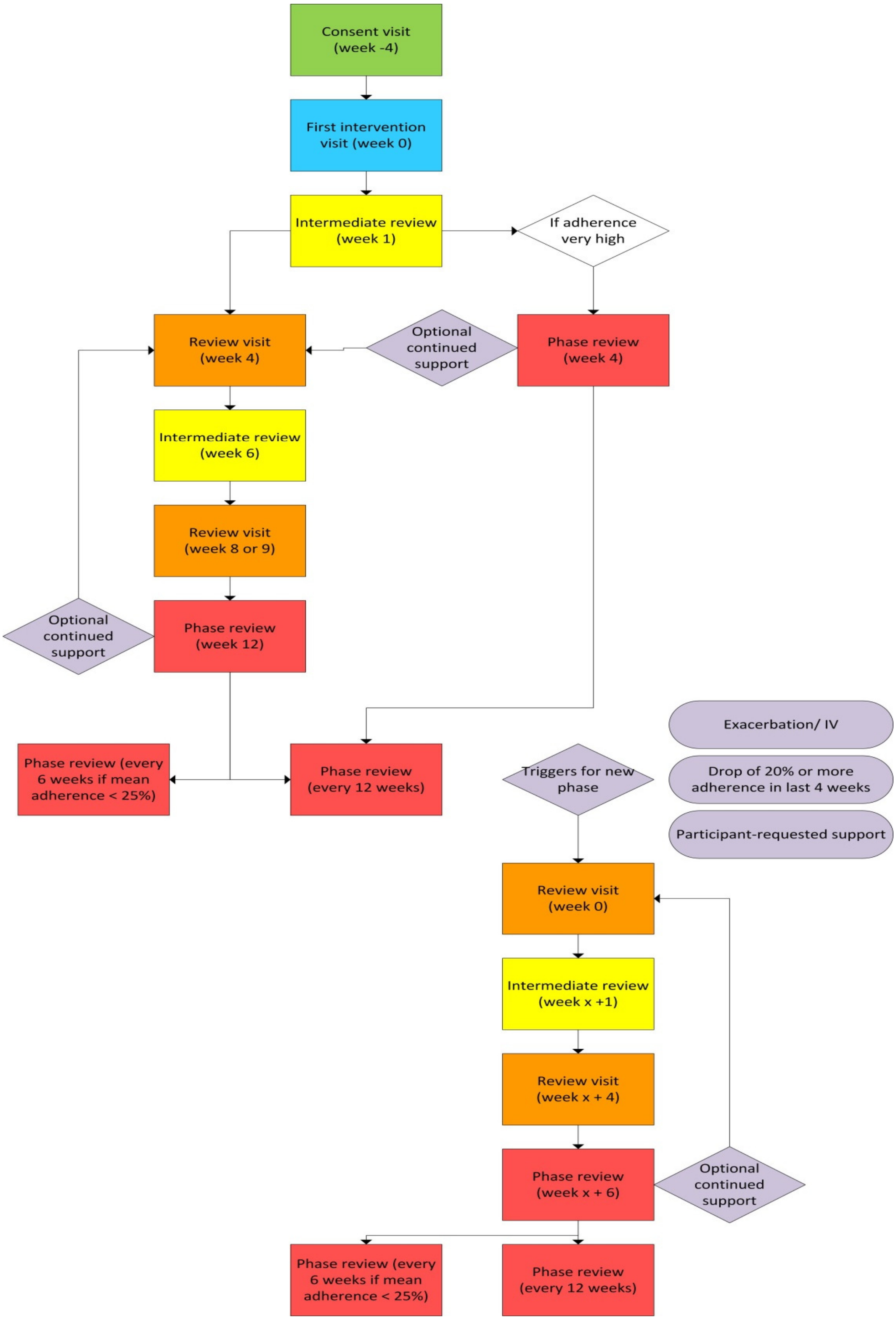

Figure A3 Data transfer process.

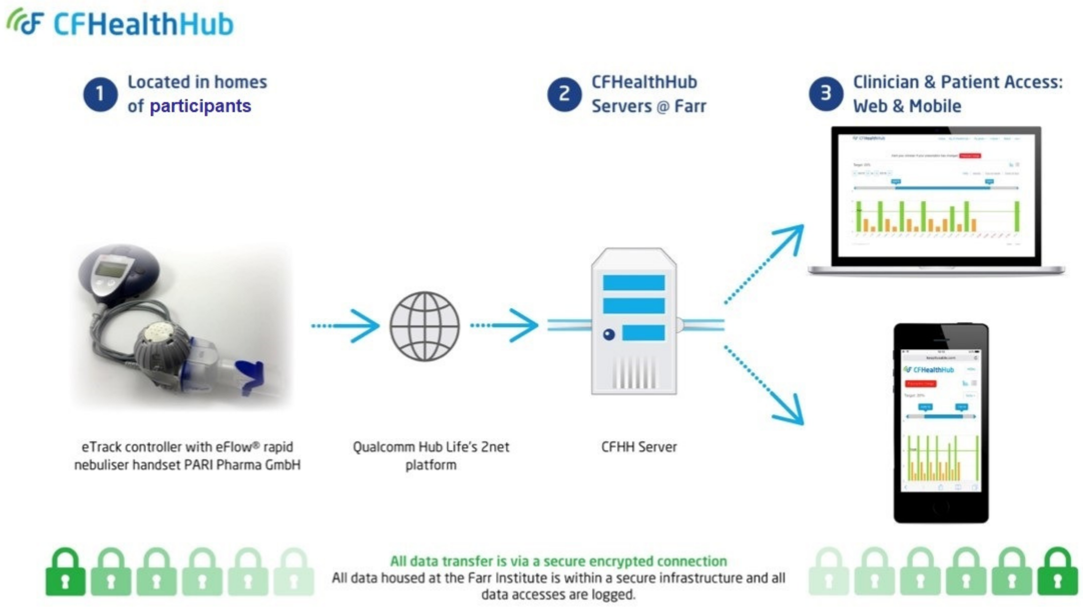

Normal pathway (adherence less than 80%)

Participants on this pathway have intervention sessions over a 12-week period. In addition to the first intervention session (at week 0) and an intermediate review (at week 1), they receive a review session at week 4, an intermediate review at week 6, a second review session at weeks 8 or 9, and a phase review at week 12. This pattern of delivery constitutes a phase. They then receive a phase review session every 12 weeks, or every 6 weeks if their objectively-measured effective adherence level is <25%.

Very high adherence pathway (adherence 80% or more)

Participants on this pathway have intervention sessions over a 4-week period. In addition to the first intervention session (at week 0) and an intermediate review (at week 1) they receive a phase review at week 4. They then receive a phase review session every 12 weeks.

Triggers

In addition to the pathways outlined above there are a number of criteria which, if met, trigger a new phase of intervention delivery. These are:

i. Participant requested support. This can be a request for additional support at a phase review in which case an additional intervention delivery period is triggered without a break, or at any other time.
ii. Additional periods of delivery are offered to participants if one or both of the following triggers occurs following the first phase review.
iii. A drop of ≥20% in objectively-measured effective adherence since the phase review.
iv. An exacerbation requiring intravenous treatment.

In any of these situations, participants are contacted, and additional support is offered. If participants agree then the triggered pathway commences with a review session at week 0, an intermediate review 1 week later, a review visit 4 weeks later, and a phase review 6 weeks later. Participants then revert back to phase reviews every 12 weeks intervals (or every 6 weeks for those with an objectively-measured effective adherence level of <25%).

Access to CFHealthHub

Participants have an individual login providing access to the CFHealthHub platform throughout the intervention. It can be accessed on a laptop or via an app available for Apple or Android devices.

Participants are encouraged to access the site regularly and are provided with a participant guide with instructions on how to access and information about what to find where.

Intervention modules

The CFHealthHub contains a number of distinct modules each of which focuses on a different aspect using a range of specific behaviour change techniques (described using the definitions in the behaviour change taxonomy)[11] and modes of delivery. Table A1 describes these techniques, and which aspects of the intervention were delivered using the CFHealthHub platform and which were delivered by the interventionist.

Table A1 Modules, behaviour change techniques and mode of delivery for the CFHealthHub intervention

Module	Behaviour change techniques[11]	Mode of delivery
My treatment	Information about health consequences<br>Credible source<br>Salience of consequences<br>Demonstration of the behaviour<br>Vicarious consequences<br>Self-talk	*CFHealthHub:*<br>• Information about CF, the need for treatment, how each treatment works and the importance of adherence<br>• Information presented in a variety of ways though written text, patient stories, 'talking heads' and animation videos, with links to external content including Cochrane reviews<br>• Range of different credible information sources including people CF, clinicians, links to scientific papers<br>*Interventionist:*<br>• Interventionist introducing and highlighting relevant content on CFHealthHub<br>• Interventionist eliciting self-talk through discussion of motivation
Self-monitoring	Self-monitoring of behaviour<br>Adding objects to the environment (CFHealthHub)	*CFHealthHub*<br>• Charts and tables of objective adherence data presented within CFHealthHub<br>*Interventionist*<br>• Introducing and explaining charts and tables to participants
Confidence building	Demonstration of behaviour<br>Focus on past success	*CFHealthHub*<br>• 'Talking heads' videos of coping stories within CFHealthHub<br>*Interventionist*<br>• Interventionist encouraging focus on periods of higher adherence on charts

Goal setting & review	Goal setting (behaviour)<br>Feedback on behaviour<br>Discrepancy between current behaviour and goal<br>Review behavioural goals<br>Graded tasks<br>Social reward	*CFHealthHub*<br>• Indication of goal line on charts of adherence<br>• Visual indication of goal met on CFHealthHub<br>• (Optional) weekly push notifications indicating whether goal was met<br>• (Optional) reward messages sent when goal met<br>*Interventionist*<br>• Discussion and agreement of goals with interventionist<br>• Review of goals<br>• Suggested steady increase in goal as improvements are made<br>• Feedback and social reward on progress
Treatment Plan	Action planning<br>Habit formation<br>Prompts/cues	*CFHealthHub*<br>• Action planning tool and storage within CFHealthHub<br>*Interventionist*<br>• Help to focus on identifying consistent cues and linking to behaviour (habit formation)<br>• Discussion and identification of appropriate cues - and how to add to the environment (if necessary)
Problem-solving	Problem solving<br>Restructure the physical environment<br>Self-talk<br>Social support (practical)<br>Instruction on how to perform the behaviour<br>Demonstration of the behaviour<br>Behavioural practice/rehearsal	*CFHealthHub*<br>• Solution bank within CFHealthHub (including advice to problem solve, restructure the physical environment, engage social support)<br>• Coping planning, Day planner and Party planner tools and storage within CFHealthHub<br>• Videos demonstrating correct use of nebulisers within CFHealthHub<br>*Interventionist*<br>• Tailored problem solving guided by interventionist<br>• Support to create Day plans/Party plans where appropriate<br>• Support to construct if-then coping plans including identifying self-talk where appropriate

CF, cystic fibrosis

Tailoring and personalisation

The CFHealthHub intervention is not one-size-fits-all and is designed to be tailored and personalised so that it can best meet the needs of a wide range of participants. While the entire content of the CFHealthHub website is available for participants to browse, tailored aspects are emphasised or added into a specific personal 'favourites' area called 'My Toolkit'. Table A2 describes the ways in which the intervention is tailored.

Table A2 Tailoring of the CFHealthHub intervention

**Tailored component**	**How non-tailored components are accessed**	**How version is determined**
Contents of 'My treatment' and 'Problem-solving' focus on information relevant to current prescription drugs	All generic information is available to all participants to browse<br>Information on treatments not currently prescribed are available but minimised	Prescription is entered into CFHealthHub at consent and altered whenever there is a prescription change<br>CFHealthHub automatically tailors content based on this information
Modules of 'My treatment' are selected and placed into 'My Toolkit' based on the scores on the COM-BMQ questionnaire	Participants can browse all modules of 'My treatment'	Participants responses to the COM-BMQ questionnaire are entered into CFHealthHub at consent. CFHealthHub recommends the most relevant modules based on a scoring algorithm<br>If CFHealthHub recommends >3 modules then interventionists select 3 based on the scores and their judgement based on conversations with the participant<br>Modules can be changed throughout the intervention and these are recorded via CFHealthHub
Modules of 'Problem-solving' are selected and placed into 'My Toolkit' based on the barriers identified in consultations with the interventionist	Participants can browse all modules of 'Problem-solving'	Interventionists can select modules of problem-solving content based on the barriers identified in consultations<br>Modules can be changed throughout the intervention and these are recorded via CFHealthHub

'Talking heads' videos are selected to match key participant characteristics and placed into 'My Toolkit'. This is optional	Participants can browse the entire 'talking heads' video library	Interventionists can select relevant videos that match key characteristics of the participant (e.g. age, gender, occupation, life role, problems experienced)<br>Videos can be changed throughout the intervention and these are recorded via CFHealthHub
Goal-setting and review and Treatment planning are only utilised for participants who are motivated (want to) take more treatment<br>Participants with very low motivation do not receive these parts of the intervention. Instead they spend more time focusing on the content of 'My treatment' and relationship building with the interventionist	Participants can choose to set goals and make plans at any point in a consultation or by contacting the interventionist	Very low motivation is determined by a combination of a low motivation score on the COM-BMQ motivation item and discussion with the participant in a consultation<br>The identification of very low motivation is recorded where this applies

COM-BMQ, COM-B Beliefs about Medicines Questionnaire.

A number of features of CFHealthHub are individually personalised for each participant. These are described in table A3.

Table A3 Personalisation of the CFHealthHub intervention

Personalised component	How personalisation is achieved
Graphs and charts show personal data	Participants eTrack nebuliser collects and send adherence data to CFHealthHub via the 2net Hub for display
Target line on graph	Participants determine their adherence goal in consultation with the interventionist. This is displayed on their charts
Plans	Participants make individual plans based on discussions with the interventionist. These are made using the tools within CFHealthHub and recorded in 'My Toolkit'. New plans can be added and CFHealthHub records all plans for each participant

Home page	Participants can select an image to display on their home page from a default selection, or can upload their own image
Notifications	Participants can optionally choose to receive personalised notifications via the CFHealthHub app. These send a message to let the participant if they have met their goal in the previous week or an encouraging messaging to keep going if they did not
Reminders	Participants can optionally choose to receive reminders via the CFHealthHub app. These send a reminder message if the participant has not accessed their CFHealthHub account for a period of 2 weeks
Reward messages	Participants can optionally choose to receive reward messages via the CFHealthHub app. These send a reward message if the participant has met their goal in the last week, 2 weeks or month

Types of intervention visit

Broadly, the intervention visits all have the same aim, which is to enable participants to look at their data, reflect on why adherence is important, set goals to increase their adherence and make plans as to how they will achieve these, and problem-solve any barriers that are likely to get in the way. However, the intervention visit types do differ somewhat in their set-up, focus and how in-depth they are, as follows. Detailed information about the structure of the delivery for each type of session is provided in the intervention manual and the relevant worksheets.

First Intervention visit

This session always happens face-to-face, although this can be in a hospital/clinic setting or at home. It lasts between 40 and 60 minutes. It is the first time that the participant accesses the CFHealthHub platform and sees their data. Interventionists must prepare for this session by entering the data from the COM-BMQ screening tool and checking that data are coming through to CFHealthHub from the nebuliser.

The key things that happen in this session are:

- Participant receives their log-in details and accesses CFHealthHub
- Participant (optionally) downloads the CFHealthHub app onto their smartphone

- Modules covered for all:
    - My treatment
    - Self-monitoring
    - Confidence building
- Modules covered for those who want to increase their treatment adherence (sufficiently motivated)
    - Goal setting
    - Treatment plan
    - Problem-solving

Intermediate review

The intermediate review is a short session that is designed to trouble-shoot 'quick' and easy to solve problems (e.g. an action plan that isn't working). It is normally delivered by telephone and lasts 5 to 15 minutes. The review is less structured than other visits.

Ad-hoc review

This follows the same structure as the intermediate review but is delivered where there is unplanned face-to-face contact with a participant (e.g. in clinic).

Review visit

This session normally last 30 to 45 minutes and can be delivered face-to-face or by telephone. The session focuses on the data and what has happened in terms of adherence since the last visit. The precise focus will vary depending on the individual participant, e.g. a session with a participant who has met their goal would have a different focus to one with a participant who has not met their goal (or did not set one).

Broadly thought, the session covers the following modules:

- My treatment
- Self-monitoring
- Confidence building
- Goal setting and review
- Treatment plan
- Problem-solving

Phase review

The focus of this appointment is to facilitate reflection on progress since the intervention (or the current phase of delivery) began and to consider whether continued support is required or whether the participant wishes to manage their adherence independently. Ideally this should be delivered face-to-face but can be delivered by telephone. It normally lasts 20 to 30 minutes.

It covers the following modules:

- My treatment
- Self-monitoring
- Confidence building
- Problem-solving

Fidelity of intervention delivery

Fidelity of delivery was assessed throughout the delivery of the intervention to ensure that interventionists continued to deliver the intervention as specified in the manual and training (assessment of drift). Two reviewers independently assessed a purposive sample of audio-recordings and worksheets associated with the delivery of intervention sessions with participants (first intervention session, review and phase review) using a scoring sheet that was developed and piloted during the feasibility trial.

Sessions were selected to represent a range of different sites, types of sessions with particular focus on interventionists who:

- Had initially failed any of their certification assessments
- Had high withdrawal rates (more than two participants withdrawn from the interventionist contact)
- Had submitted <80% audio-recorded sessions from those participants who provided consent for them to be recorded
- Had completed a lower than expected number of intervention visits and/or had fewer than average action and coping plans recorded in CFHealthHub

Metrics for fidelity of intervention delivery

There were 32 interventionists and a total of 213 fidelity of delivery assessments conducted during the randomised controlled trial.

110 assessments were assessed to explore drift in fidelity over the duration of the trial and a pass mark threshold of 80% was set was drift assessments. Of all paired assessments during the randomised controlled trial there was 97.2% agreement when comparing pass/fail decisions at the 80% threshold (207 of 213 assessments in agreement).

Intervention fidelity delivery scores are summarised by session type in table A4 and by site in table A5. Delivery of the intervention had very good fidelity (overall fidelity by site range 79–97%) with only one site not achieving over the mean threshold (>80%) on drift assessments.

Table A4 Intervention fidelity delivery score summaries by session type

Session type	Assessment*	N	Median	Interquartile range
First intervention visit	First fidelity	27	97.2	92.3, 100.0
	Fidelity reassessment	1	98.6	98.6, 98.6
	Drift	29	95.8	93.1, 97.2
Review	First fidelity	30	92.6	87.0, 98.1
	Fidelity reassessment	9	96.3	94.4, 96.3
	Drift	47	92.6	90.2, 96.3
Phase review	First fidelity	30	94.4	91.7, 97.2
	Fidelity reassessment	6	97.2	93.1, 99.3
	Drift	34	94.4	91.7, 97.2

*Reasons for assessment, with multiple reasons possible: certification (97), reassessment after failed certification (36), high withdrawal rate (18), insufficient audio-recorded sessions (37), fewer than expected intervention visits or action/coping plans created (82), random to ensure total assessment sample ≥20% of all interventionist visits (9).

Table A5. Overall intervention fidelity scores by site

Site	Fidelity score	Site (continued)	Fidelity score (continued)
1	92.4	11	93.2
2	93.2	12	92.4
3	96.6	13	94.8
4	89.9	14	94.9
5	78.7	15	87.4
6	94.0	16	92.8
7	89.3	17	94.3
8	86.6	18	94.7
9	98.3	19	95.0
10	90.5		

References

1. Michie S, van Stralen MM, West R. The behavior change wheel: a new method for characterising and designing behavior change interventions. *Implement Sci* 2011;6:42.
2. Arden MA, Hutchings M, Whelan P, *et al.* Development of an intervention to increase adherence to nebuliser treatment in adults with cystic fibrosis: CFHealthHub. *Pilot Feasibility Stud* 2021;7:1.
3. Bandura A. Social cognitive theory of self-regulation. *Organ Behav Hum Decis Process* 1991;50:248–87.
4. Carver CS, Scheier MF. Control theory: a useful conceptual framework for personality–social, clinical, and health psychology. *Psychol Bull* 1982;92:111–35.
5. Gardner B. A review and analysis of the use of 'habit' in understanding, predicting and influencing health-related behaviour. *Health Psychol Rev* 2015;9:277–95.
6. Horne R, Weinman J. Patients' beliefs about prescribed medicines and their role in adherence to treatment in chronic physical illness. *J Psychosom Res* 1999;47:555–67.
7. Lally P, van Jaarsveld CHM, Potts HWW, Wardle J. How are habits formed: modelling habit formation in the real world. *Eur J Social Psychol* 2009;40:998–1009.
8. Wood W, Neal DT. The habitual consumer*. J Consum Psychol* 2009;19:579–92.
9. Lally P, Wardle J, Gardner B. Experiences of habit formation: a qualitative study. *Psychol Health Med* 2011;16:484–9.
10. Rothman AJ, Sheeran P, Wood W. Reflective and automatic processes in the initiation and maintenance of dietary change. *Ann Behav Med* 2009;38 (Suppl 1):S4–17.
11. Michie S, Richardson M, Johnston M, *et al.* The behavior change technique taxonomy (v1) of 93 hierarchically clustered techniques: building an international consensus for the reporting of behavior change interventions. *Ann Behav Med* 2013;46:81–95.

APPENDIX B The choice of adherence measure

In the trial, to reflect effective medication use, adherence was calculated as normative (effective) adherence using objective data from Weeks 3–52 as the outcome and Weeks 1&2 as the "baseline". Objectively-measured effective adherence was adopted as an outcome measure because it better reflects the effectiveness of medication use in comparison to simply calculating percent adherence according to an agreed regimen between adults with cystic fibrosis (CF) and their clinical team, as we have detailed elsewhere.[1-3] The calculation of objectively-measured effective adherence involves numerator adjustment (capping daily maximum nebuliser use at 100%) and denominator adjustment (to define the minimum effective treatment regimen) according to a person's *Pseudomonas aeruginosa* status, as described in section 9.2.1 of the statistical analysis plan (available as supplementary material). For example, a person with chronic *Pseudomonas aeruginosa* infection should be on at least a nebulised muco-active agent and an antibiotic (i.e. three daily doses). If a person with chronic *Pseudomonas aeruginosa* infection only agreed to use nebulised dornase alfa once daily, adherence levels in outcome calculation will use the denominator of three daily doses. If that person was on aztreonam thrice daily and hypertonic saline twice daily, no denominator adjustment will be carried out because denominator adjustment only applies for less than ideal regimen. In particular, the denominator adjustment is important because there is a wide variation in the prescription of inhaled therapies between different centres.[4] By standardising the denominator given the person's clinical characteristics in calculating objectively-measured effective adherence, it is ensured that an increase in percentage adherence is due to an increase in nebuliser use (i.e. increase in the numerator) rather than simply due to a reduction in agreed prescriptions (i.e. decrease in the denominator).

It is important to distinguish the concept of standardisation for effectiveness used as an outcome measure from individualised feedback to participants. Objectively-measured effective adherence allows standardisation based on randomised controlled trial evidence of what treatment is likely to work. Individualised target setting between clinical teams and people with CF continued to be informed by both considerations of effective treatments and considerations of what the person feels they wish to aim for. On occasions within the trial, clinicians and participant may have agreed on regimens that exceed the minimum number of doses that would be considered effective given a participant's characteristics. Since effective adherence denominator adjustments are intended simply to ensure minimum level of effectiveness, no adjustments were necessary in the case of these participants. That is to say the denominator adjustment was a strategic instrument to ensure minimal level of effectiveness is being reflected in the calculation of percent adherence.

Figure B1 Data display of CFHealthHub.

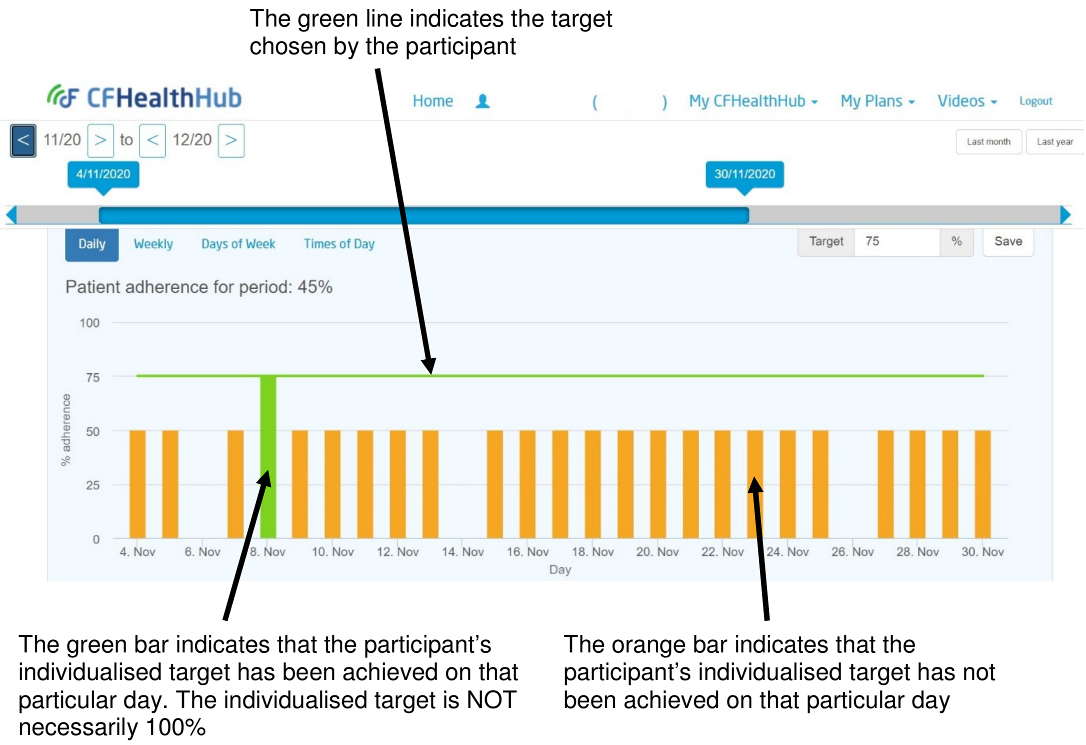

CFHealthHub interventionists were trained to continue to record prescriptions that fulfil the minimum effective dose requirement. In the example of figure B1, the participant is a person with chronic *Pseudomonas aeruginosa* infection who aimed for three daily doses of aztreonam but muco-active agent was not part of their agreed treatment target. Data feedback within CFHealthHub captured individualised targets by displaying a target reflecting the treatment(s) that the participant chose to aim for. If they used all three daily doses of aztreonam, their effective adherence on the day would be 3/(3 + 1) = 75%. This personalised decision would appear as the target set on CFHealthHub in the form of the green line on the graph (at 75% adherence). When fewer than three daily doses were used, the daily adherence bar will be displayed in orange (for example on 23 November). When three daily doses were used, which met their individualised target though it did not achieve an effective adherence of 100%, the daily adherence bar will be displayed in green (for example on 08 November). Therefore, the data display of CFHealthHub feeds back the individualised target. In this example, the agreed prescribed regimen was still recorded as three daily doses of aztreonam and a daily dose of dornase alfa, though the participant was only aiming for three daily doses of aztreonam. By lowering the treatment target rather than reducing prescribed doses, any deviation from effective targets will still be visible on CFHealthHub. It is important to emphasise that this data

display was produced in collaboration with people with CF and reflected their preference for representing individualised targets within the context of the evidence base around optimally effective treatments.

The primary analysis of adherence for randomised clinical trial reporting was standardised using the concept of adherence to a regimen considered to be effective. Any deviation from the guidance to enter effective prescription into CFHealthHub or errors that were made based on a lack of awareness, for example of *Pseudomonas aeruginosa* status, were corrected for in the analysis which ensured that adherence at all sites and for all participants were being compared on an equal basis, i.e. effective adherence. That is to say the analysis of participant data for someone with chronic *Pseudomonas aeruginosa* infection only using inhaled antibiotic recognises it to be a regimen not considered to be maximally effective by international consensus.[5-7] Thus denominator adjustment in this case would ensure that the adherence level analysed against the primary outcome of exacerbation would be not be 100%, but would be capped to a maximum of 75%, as in the example of figure B1. Without such standardisation, a person with more effective nebuliser use would not be identified in the calculation of percent adherence. Rigour around effectiveness is an important element in understanding the relationship between adherence and health outcomes. For example, without denominator adjustment, a person with chronic *Pseudomonas aeruginosa* infection using an average daily dose of one inhaled antibiotic and one dornase alfa but prescribed a total of three daily doses would have adherence of 67% yet a similar person using just a daily dose of dornase alfa but prescribed a total of one daily dose would have adherence of 100%.

By using objectively-measured effective adherence as the method of calculating adherence, we can be confident that an increase in percent adherence reflects more effective medication use. It is important, given the use of effective adherence, to highlight that participants with chronic *Pseudomonas aeruginosa* infection were equally distributed between intervention and usual care. Yet usual care had slightly higher prescribed daily doses (mean 3.1 vs 2.9, see table 2 of main manuscript for breakdown of prescribed doses), meaning that the denominator adjustment would have reduced effective adherence among intervention participants to a greater extent compared to usual care. That is to say the use of objectively-measured effective adherence if anything, would bias against the intervention group.

Figure B2 Mean inhaled doses taken per week.

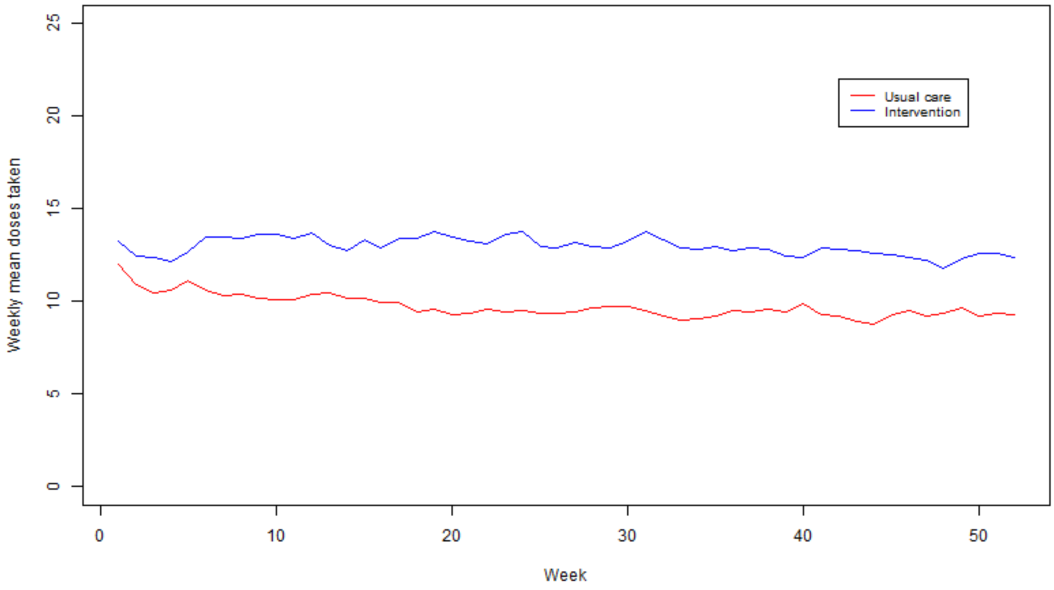

Figure B3 Weekly mean objectively-measured effective adherence.

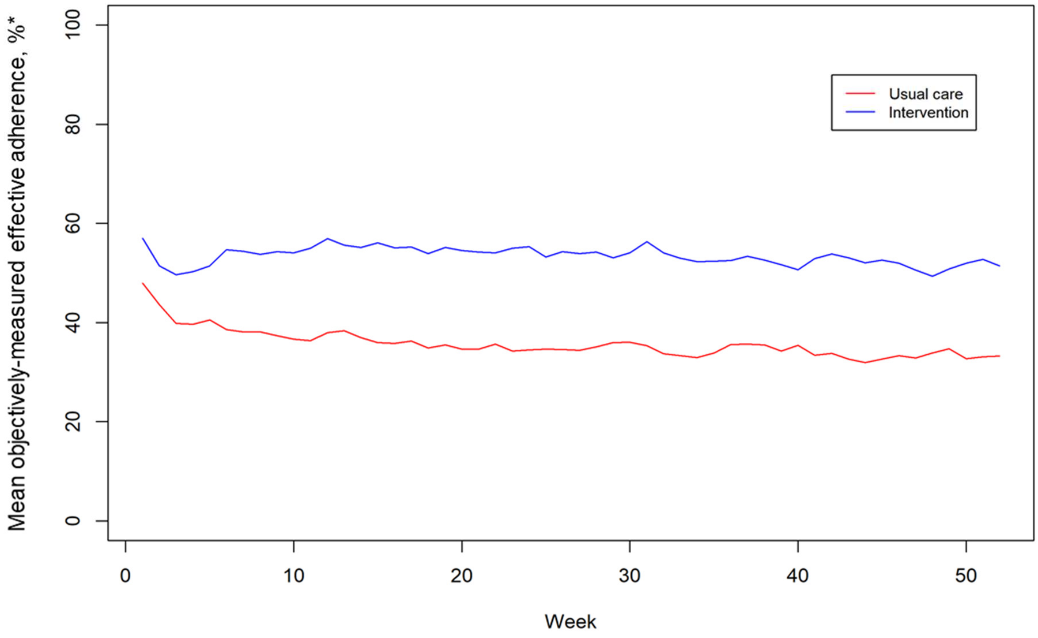

It is reassuring that as the intervention was delivered, a clear between-group divergence in the mean inhaled doses emerged (figure B2). That is to say the intervention group used more doses of nebulisers, which mirrors the divergence in objectively-measured effective adherence (figure B3). Therefore, the difference in calculated percent effective adherence was driven by the number of doses taken (numerator) among intervention participants rather than prescription (denominator) adjustments. The fact that the absolute number of doses between intervention and control diverges indicates that the use of objectively-measured effective adherence is capturing a difference in absolute treatment use between intervention and control.

The percent objectively-measured adherence without any adjustments also mirrors the difference observed with objectively-measured effective adherence, as shown in table B1.

Table B1 Objectively-measured adherence, by unadjusted and effective calculations

	**Usual care**	**Intervention**
Baseline (weeks 1 & 2)	N = 295	N = 293
Unadjusted adherence		
Mean (SD)	48.2 (34.4)	56.4 (32.4)
Median (IQR)	50.0 (14.3, 81.0)	61.3 (28.6, 85.7)
Effective adherence		
Mean (SD)	45.5 (34.1)	54.1 (33.0)
Median (IQR)	42.9 (10.7, 76.4)	57.2 (25.0, 84.2)
Weeks 3 to 26	N = 301	N = 301
Unadjusted adherence		
Mean (SD)	38.0 (33.0)	56.3 (31.6)
Median (IQR)	29.0 (6.5, 68.3)	63.6 (31.4, 84.3)
Effective adherence		
Mean (SD)	35.9 (32.2)	53.7 (31.7)
Median (IQR)	25.9 (6.2, 61.6)	58.7 (26.8, 81.4)
Weeks 27 to 52	N = 282	N = 288
Unadjusted adherence		
Mean (SD)	35.4 (32.7)	55.2 (32.6)
Median (IQR)	27.6 (4.0, 64.6)	64.0 (23.3, 83.0)
Effective adherence		
Mean (SD)	33.2 (31.7)	51.9 (32.6)
Median (IQR)	24.4 (3.5, 59.8)	56.2 (22.5, 81.4)

IQR, interquartile range; SD, standard deviation.

References

1. Hoo ZH, Curley R, Campbell MJ, Walters SJ, Hind D, Wildman MJ. Accurate reporting of adherence to inhaled therapies in adults with cystic fibrosis: methods to calculate “normative adherence”. *Patient Prefer Adherence* 2016;10:887–900.
2. Hoo ZH, Curley R, Walters SJ, Campbell MJ, Wildman MJ. Exploring the implications of different approaches to estimate centre-level adherence using objective adherence data in an adult cystic fibrosis centre - a retrospective observational study. *J Cyst Fibros* 2020;19:162–7.
3. Hoo ZH, Totton N, Waterhouse S, *et al*. Real-world adherence among adults with cystic fibrosis is low – a retrospective analysis of the CFHealthHub digital learning health system. *Chest* 2021 Jun 26 [Epub ahead of print]
4. UK Cystic Fibrosis Registry. Annual data report 2018. Published August 2019. https://www.cysticfibrosis.org.uk/the-work-we-do/uk-cf-registry/reporting-and-resources. [Accessed 29 April 2021].
5. Mogayzel PJ Jr, Naureckas ET, Robinson KA, *et al.* Cystic fibrosis pulmonary guidelines. Chronic medications for maintenance of lung health. *Am J Respir Crit Care Med* 2013;187:680–9.
6. Castellani C, Duff AJA, Bell SC, *et al.* ECFS best practice guidelines: the 2018 revision. *J Cyst Fibros* 2018;17:153–78.
7. Villanueva G, Marceniuk G, Murphy MS, Walshaw M, Cosulich R; Guideline Committee. Diagnosis and management of cystic fibrosis: summary of NICE guidance. *BMJ* 2017;359:j4574.

APPENDIX C Participant recruitment

As is discussed in appendix D, "baseline adherence" of the participants may have been exaggerated by novelty effect and white coat adherence. Nonetheless, we also acknowledge that the study may have recruited a convenience sample that was more focused on adherence compared to the general cystic fibrosis (CF) population. The baseline median adherence of the participants was 52% whereas real world median adherence among adults with CF has been reported as closer to 35%.[1,2] Another observation supporting the contention that a more engaged sample was recruited in this trial is the intravenous (IV) antibiotic rejection rate for exacerbations among this sample of around 5% (see table S3 footnote), which is four-fold lower than in real-world dataset where the IV rejection rate is around 20%.[3]

In the CONSORT diagram (figure 2 of main manuscript), we report that 3510 adults with CF were screened and 608 were recruited. The discrepancy between screening and recruitment was driven by a decision to prioritise rapid recruitment because more than two-thirds of large publicly funded trials in the United Kingdom (UK) failed to recruit to time and target.[4] As such, all adults with CF in participating centres were screened using data from the UK CF registry and investigators may have also first approached those they thought would be most amenable to participating. Once a centre reached its recruitment target (around 35 participants per centre), recruitment for the centre would be closed and a large proportion of other screened adults (each centre would have screened on average 150–200 adults) would be unable to participate. This strategy has enabled us to recruit 608 participants in just 8 months (even though not all centres open for recruitment at the same time), which is ahead of the recruitment target.

Although a biased sample that was more focused on adherence may have been recruited as the result of the recruitment strategy, it is important to consider the direction of any resultant bias. As is discussed in appendix D, there is a ceiling effect associated with high baseline adherence.[5,6] It may follow that scope for improvement in adherence in our trial was curtailed in the intervention arm by ceiling effects associated with high baseline adherence and nearly 30% of the participants having baseline adherence >75%. Therefore, any bias associated with the recruitment strategy would be towards null effect and the overall adherence difference of adjusted mean difference of 9.5 percentage points (95% confidence interval 8.6, 10.4) may have been an under-estimate.

References

1. Hoo ZH, Curley R, Walters SJ, Campbell MJ, Wildman MJ. Exploring the implications of different approaches to estimate centre-level adherence using objective adherence data in an adult cystic fibrosis centre – a retrospective observational study. *J Cyst Fibros* 2020;19:162–7.

2. Daniels T, Goodacre L, Sutton C, Pollard K, Conway S, Peckham D. Accurate assessment of adherence: self-report and clinician report vs electronic monitoring of nebulizers. *Chest* 2011;140:425–32.

3. Hoo ZH, Bramley NR, Curley R, *et al.* Intravenous antibiotic use and exacerbation events in an adult cystic fibrosis centre: a prospective observational study. *Respir Med* 2019;154:109–15.

4. Campbell MK, Snowdon C, Francis D, *et al.* Recruitment to randomised trials: strategies for trial enrollment and participation study. The STEPS study. *Health Technol Assess* 2007;11:iii,ix–105.

5. Sieben A, Bredie SJH, Luijten JCHBM, van Laarhoven CJHM, van Dulmen S, van Onzenoort HAW. Prior medication adherence of participants and non participants of a randomized controlled trial to improve patient adherence in cardiovascular risk management. *BMC Med Res Methodol* 2019;19:95.

6. Lin EHB, Von Korff M, Ciechanowski P, *et al.* Treatment adjustment and medication adherence for complex patients with diabetes, heart disease, and depression: a randomized controlled trial. *Ann Fam Med* 2012;10:6–14.

APPENDIX D Between-group imbalance and baseline adherence

Table 2 of the main manuscript suggests there may be some imbalance between usual care and intervention groups at baseline. The intervention group was around 1 year older (mean age 31.1±10.6 years versus 30.3±10.8 years) yet percent predicted forced expiratory volume in one second (FEV_1) was higher by around 2 (60.7±23.5 versus 58.3±22.6) and annual intravenous (IV) antibiotics use was lower by around 3 days (24.2±27.9 days versus 27.7±33.0 days). This may suggest that the intervention participants had slightly better lung health at baseline, which may be due to higher adherence prior to recruitment. Indeed, there is also imbalance of "baseline adherence", that is the objectively-measured effective adherence level measured in the first two weeks post randomisation, which was around 9% in favour of the intervention group (54.1±33.0% versus 45.5±34.1%).

In this section, we deal with the following five issues:

1) Explain how the randomisation process could result in baseline imbalance despite 608 participants being randomised
2) Explore the likely impact of age on the baseline adherence
3) Provide analyses which explore the adherence trajectory for intervention versus usual care after minimising the imbalance of baseline adherence
4) Explore the impact of baseline imbalance in terms of the direction of bias on the observed effect size
5) Explore how these limitations can be minimised to make future trials more efficient

1) The randomisation process

The imbalance in baseline parameters is likely due to a randomisation process which involved two levels of stratification (centre and past-year IV days, as described in Section 9.1 of the protocol [available as supplementary material]) which limits the block size. Each centre recruited around 35 participants and the aim was to achieve approximately similar numbers of usual care and intervention participants in each centre, so that the centre interventionists were not overwhelmed by excess number of intervention participants. Thus the play of chance is not acting on 608 participants but is acting on a maximum block size of 35 with two levels of stratification to randomise participants into usual care and intervention; i.e. the play of chance is constrained by limited block size.

2) Impact of age on the baseline adherence

The intervention group was around 1 year older. The adherence imbalance at the initial part of the trial may in part be influenced by differences in the proportion of participants according to age categories. Multiple studies have demonstrated a strong association between the age categories (16-18 years, 19–25 years, 26–34 years, ≥35 years) and adherence levels.[1,2] The usual care arm has an excess of younger participants with lower adherence and the intervention arm has an excess of older participants with higher adherence (figure D1). There were 27 usual care and 17 intervention participants aged 16–18 years, where the mean baseline adherence for 44 participants was 31%. There were 75 usual care and 91 intervention participants aged ≥35 years, where the mean baseline adherence for 166 participants was 62%. By plotting adherence according to age categories, the effect of age imbalance at the start of the trial is clearer. There is less adherence imbalance at the start of the trial when participants were grouped by age (figure D1) except for the few participants aged 16–18 years (n=44, 7%). Some of the baseline adherence imbalance following age stratification may be due to the transient effect of enhanced white coat adherence in the intervention group who were aware from Day 1 that a planned 3-week meeting with interventionists to review their data would occur. This is consistent with the behaviour change technique of feedback used as part of the intervention and contrasts with the usual care group who were aware that adherence measurement would simply be used for research and neither fed back nor reviewed by interventionists.

Figure D1 Adherence curves according to different age categories.

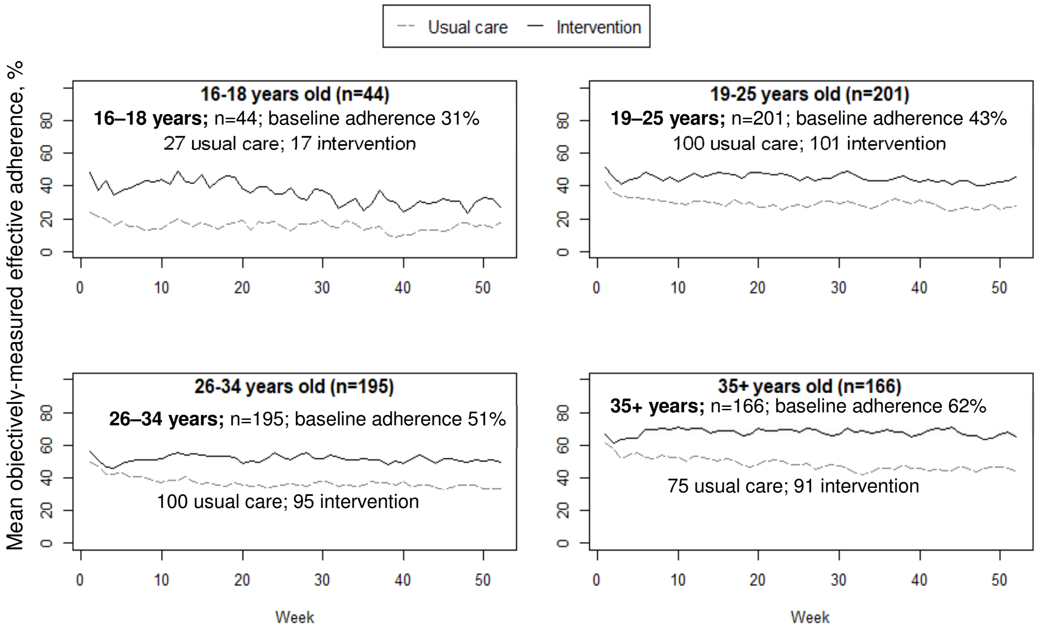

Figure D2 Adherence curves, and mean between-group difference in objectively-measured effective adherence, according to baseline adherence. CI, confidence interval; Int-UC, intervention minus usual care.

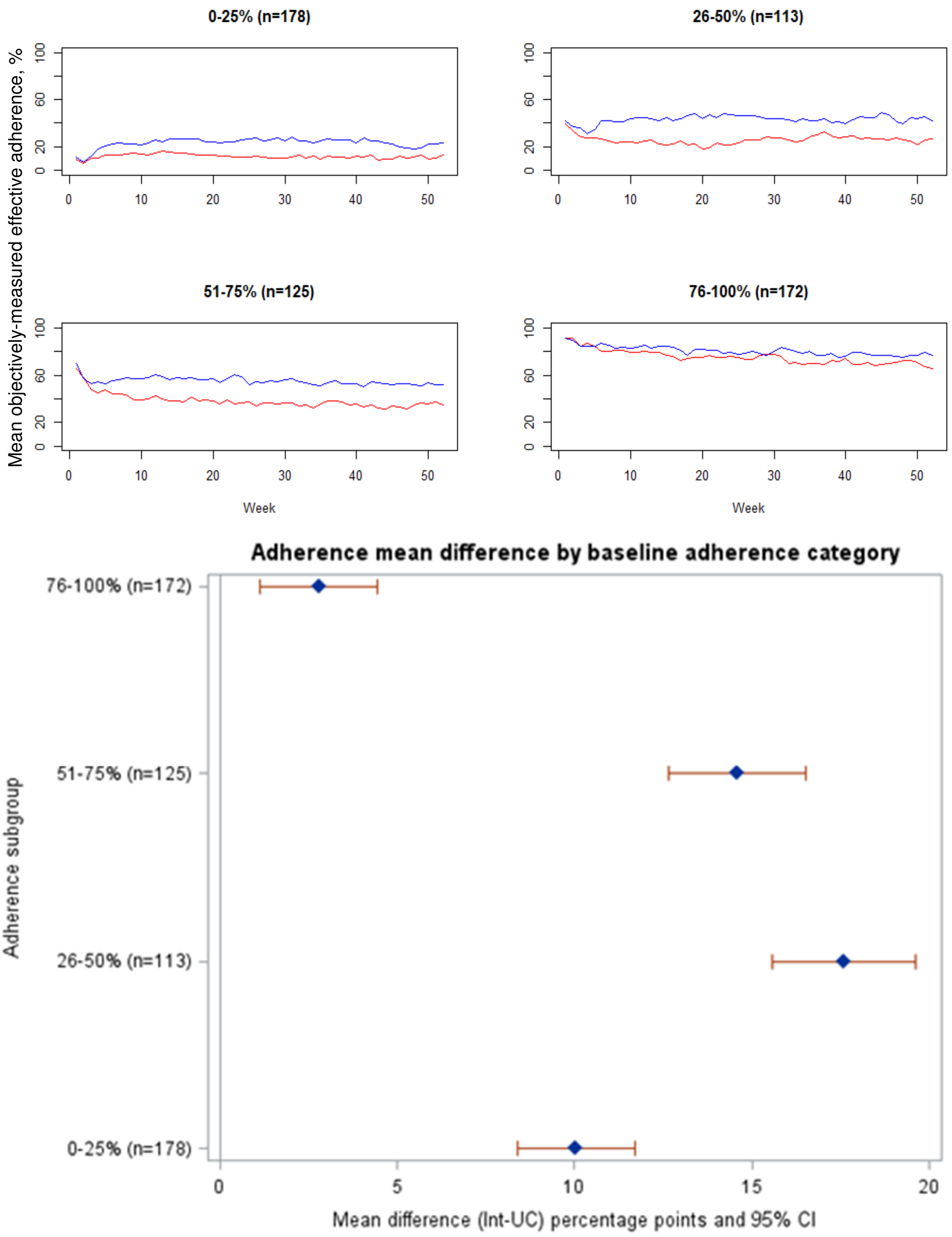

3) Explaining the adherence trajectory for intervention versus usual care

Trial participants in both arms had never previously been monitored with data-logging nebulisers and would likely be susceptible to novelty effect and whitecoat adherence at the initial part of the trial,[3-6] with the consequence that adherence in the first two weeks was unrepresentative of their steady-state adherence. Of note, the baseline adherence imbalance was discussed in the previous section and was unrelated to novelty effect or white coat adherence. It is known that novelty effect and whitecoat adherence are relatively short-lived[3-6] and this is reflected in the initial sharp adherence decline for both arms seen in the study (figure 3 of main manuscript; figure D1). Among control participants who did not receive any intervention, this decline continued over the next 12 weeks to around 35%, which is the real-world objective adherence level for inhaled therapies among adults with CF,[7,8] and stayed at this level until the end of the trial. In the intervention group, the initial rate of decline was similar to the controls until the behavioural-change intervention started from Week 3 and adherence subsequently improved. It is also important to note that the separation in adherence curves between intervention and usual care participants occurred regardless of baseline adherence when curves were plotted by adherence categories (figure D2).

4) The impact of baseline imbalance on the direction of bias

There is a ceiling effect associated with high adherence.[9,10] Indeed, subgroup analysis according to baseline adherence (figure D2) indicates minimal end of study between-group difference in objectively-measured effective adherence among those with baseline adherence >75%. It is therefore likely that a preponderance of high adherers among the intervention group would bias the overall adjusted adherence results towards null effect, i.e. the overall adjusted mean difference in objectively-measured effective adherence of 9.5 percentage points (95% confidence interval 8.6, 10.4) may have been larger had those with baseline adherence >75% been excluded.

5) How these limitations can be minimised to make future trials more efficient

As discussed in the main manuscript, the measurement of “baseline adherence” in the first two weeks post randomisation is a limitation of the trial. It would have been ideal to obtain an understanding of the study participants’ actual baseline adherence by measuring adherence over longer periods prior to randomisation, which may allow white coat adherence among adults using data-logging nebulisers for the first time to wear off. The decay of usual care participants’ adherence to baseline took approximately 12 weeks, suggesting the importance

of providing objective adherence monitoring technology to participants for at least 12 weeks before baseline adherence is captured. This would impact time scales for an adherence trial and the funding envelope requested. In our subsequent trials, we plan to nest the evaluation of adherence interventions within a digital learning health system (ISRCTN14464661) so that baseline adherence can be understood prior to randomisation. This has a number of benefits, including recruiting participants in whom adherence can be seen to improve from baseline (effectively removing the impact of whitecoat adherence) and greater efficiency by avoiding the recruitment of potential participants with maximal adherence at baseline.

References

1. Quittner AL, Zhang J, Marynchenko M, *et al.* Pulmonary medication adherence and health-care use in cystic fibrosis. *Chest* 2014;146:142–51.
2. Eakin MN, Bilderback A, Boyle MP, Mogayzel PJ, Riekert KA. Longitudinal association between medication adherence and lung health in people with cystic fibrosis. *J Cyst Fibros* 2011;10:258–64.
3. Podsadecki TJ, Vrijens BC, Tousset EP, Rode RA, Hanna GJ. "White coat compliance" limits the reliability of therapeutic drug monitoring in HIV-1-infected patients. *HIV Clin Trials* 2008;9: 238–46.
4. McConville A, Noser AE, Clements MA, Patton SR. Mealtime insulin BOLUS score increases prior to clinic visits in youth with type 1 diabetes. *BMJ Open Diabetes Res Care* 2020;8:e001348.
5. Modi AC, Ingerski LM, Rausch JR, Glauser TA, Drotar D. White coat adherence over the first year of therapy in pediatric epilepsy. *J Pediatr* 2012;161:695–9.e1.
6. Shin G, Feng Y, Jarrahi MH, Gafinowitz N. Beyond novelty effect: a mixed-methods exploration into the motivation for long-term activity tracker use. *JAMIA Open* 2019;2:62–72.
7. Hoo ZH, Curley R, Walters SJ, Campbell MJ, Wildman MJ. Exploring the implications of different approaches to estimate centre-level adherence using objective adherence data in an adult cystic fibrosis centre – a retrospective observational study. *J Cyst Fibros* 2020;19:162–7.
8. Daniels T, Goodacre L, Sutton C, Pollard K, Conway S, Peckham D. Accurate assessment of adherence: self-report and clinician report vs electronic monitoring of nebulizers. *Chest* 2011;140:425–32.

9. Sieben A, Bredie SJH, Luijten JCHBM, van Laarhoven CJHM, van Dulmen S, van Onzenoort HAW. Prior medication adherence of participants and non participants of a randomized controlled trial to improve patient adherence in cardiovascular risk management. *BMC Med Res Methodol* 2019;19:95.

10. Lin EHB, Von Korff M, Ciechanowski P, *et al.* Treatment adjustment and medication adherence for complex patients with diabetes, heart disease, and depression: a randomized controlled trial. *Ann Fam Med* 2012;10:6–14.

Appendix E Other limitations of the trial

In the main manuscript, the limitations of using exacerbation as the primary endpoint which may bias the result towards null effect and the difficulty of discerning the trajectory of intervention effect due to a lack of pre-randomisation steady-state adherence level were discussed. In this appendix, we discuss the other limitations of the trial.

One of the potential limitations is the delivery of both behavioural change and research processes by interventionists. The intervention was delivered via CFHealthHub, which was unavailable to usual care participants. Mixed-methods process evaluation of our two-centre pilot study, which specifically addressed contamination, demonstrated negligible contamination among usual care participants.[1] Outcome data were objective measures unlikely to be biased by interventionists' data collection.[2]

Three other limitations of the trial might bias the observed results towards a null effect. First, a convenience sample was recruited with around 30% of the participants having baseline adherence >75%, a subgroup in whom an impact on outcome measures would be unlikely, rendering the trial less efficient. It is noteworthy that the intravenous (IV) antibiotic rejection rate in this trial was around 5% whereas the real-world IV rejection rate is typically four-fold higher at around 20%,[3] supporting the contention that a more engaged sample was recruited in this trial. The ceiling effect among high adherers means that the effect size would have been larger if high adherers were excluded (see appendices C and D). With this limitation, any observed difference in adherence in the trial could be considered particularly noteworthy. Since trial participants may have better health outcomes than non-participants,[4] there may also be ceiling effect on health outcomes as well as ceiling effect on adherence. If we assume the intervention is able to impact people with lower levels of adherence, the outcomes seen in this opportunistic sample might have a larger effect size in the whole population where median adherence is ~30%. Interestingly, the FEV_1 difference did not include unity in the subset of participants with adherence <25%. This further supports the assertion that focusing an adherence intervention study on participants with lower levels of adherence has the advantage of both trial efficiency and increased probability of impacting health outcomes such as FEV_1.

Second, there was a period of server downtime which affected intervention delivery. Adherence data were not lost but simply inaccessible during the downtime. Interventions were delivered over 80 weeks (9 months for recruitment) and the CFHealthHub server experienced a 43-day outage at one point, which delayed the receipt of data to the server such that the platform was inaccessible to all participants during this period. Intervention sessions would be rescheduled if adherence data were unavailable, meaning that no intervention took place during this period. The server hosting infrastructure was improved following the downtime,

reducing the likelihood of future issues. Fidelity assessments throughout the trial, which required the use of objective adherence data during sessions, showed reassuring scores of 93–97%. Given the importance of the platform for intervention delivery, unavailability would reduce the intervention effectiveness and bias the results towards null effect. However, in the spirit of intent-to-treat analysis, we did not make any adjustments to avoid over-estimating treatment effect. It is important to emphasise that periods where data transfer was delayed did not result in data loss as data were simply backed up and transferred once system transfer was restored.

Third, the trial was underpowered to detect the observed point estimate in forced expiratory volume in one second (FEV_1). Sample size calculation in table S1 showed that the trial has under 80% power to detect a 6 point difference in between-group percent predicted FEV_1. The observed between-group point estimate of 1.4 in percent predicted FEV_1 at 12 months may simply be due to chance but is within the range observed for hypertonic saline at 48 weeks.[5]

Overall, these four limitations (alongside the limitations of exacerbation as the primary outcome) reduced the trial's ability to demonstrate statistically significant improvements in lung health. The significant albeit small difference in body mass index (BMI) with the intervention versus usual care should be noted, and higher BMI has shown an association with higher FEV_1.[6] It is possible that FEV_1 improvement may emerge gradually over time with longer follow-up.

It is also possible that improvement in health outcomes may not be linearly associated with the increase in adherence; for example, there may be both a threshold effect and a ceiling effect. The relationship between improvement in treatment adherence and improvement in health outcomes among people with CF is relatively unexplored, in part because previous adherence trials did not demonstrate improved adherence. Further analyses would be performed using the ACtiF dataset to better understand the relationship between adherence to chronic therapies and health outcomes.

References

1. Hind D, Drabble SJ, Arden MA, *et al.* Feasibility study for supporting medication adherence for adults with cystic fibrosis: mixed-methods process evaluation. *BMJ Open* 2020;10:e039089.
2. Moustgaard H, Clayton GL, Jones HE, *et al.* Impact of blinding on estimated treatment effects in randomised clinical trials: meta-epidemiological study. *BMJ* 2020;368:l6802.
3. Hoo ZH, Bramley NR, Curley R, *et al.* Intravenous antibiotic use and exacerbation events

in an adult cystic fibrosis centre: a prospective observational study. *Respir Med* 2019;154:109–15.

4. Goss CH, Rubenfeld GD, Ramsey BW, Aitken ML. Clinical trial participants compared with nonparticipants in cystic fibrosis. *Am J Respir Crit Care Med* 2006;173:98–104.
5. Wark P, McDonald VM. Nebulised hypertonic saline for cystic fibrosis. *Cochrane Database Syst Rev* 2018;9:CD001506.
6. Stallings VA, Stark LJ, Robinson KA, *et al.* Evidence-based practice recommendations for nutrition-related management of children and adults with cystic fibrosis and pancreatic insufficiency: results of a systematic review. *J Am Diet Assoc* 2008;108:832–9.
